# Abstracts of papers presented at the 1999 Pittsburgh Conference

**DOI:** 10.1155/S1463924699000218

**Published:** 1999


Journal of Automated Methods & Management in Chemistry, Vol. 21, No. 6 (November–December 1999) pp. 185–210

### Abstracts of papers presented at the 1999 Pittsburgh Conference

The following 70 abstracts form Part B of three issues of *Journal of Automated Methods & Management in Chemistry* devoted to abstracts of papers presented this year at the 50th Anniversary Pittcon held in March in New Orleans, LA. The editors have selected, from over 1000 presentations, those of particular interest to the *Journal of Automatic Chemistry's* readers. For information on next year's Pittcon, contact The Pittsburgh Conference, 300 Penn Center Boulevard, Suite 332, Pittsburgh, PA 15235–5503, USA. Tel.: 412 825 3220; fax.: 412 825 3224; World Wide Web: http://www.pittcon.org.

### Enhanced selectivity for sulphur and phosphorus compounds using the pulsed flame-photometric detector (PFPD) with multiple time gates

*Donald P. Segers, Melody L. Thompson and Kenneth A. Kuhn, OI Analytical, CMS Field Products Group, 200 Chase Park South, Suite 100, Birmingham, AL 35244, USA*

The conventional flame-photometric detector (FPD) is often used in gas-chromatographic analyses for sulphur-containing compounds and phosphorus-containing compounds, e.g. pesticides. The FPD has a continuous hydrogen/air flame that simultaneously generates excited chemiluminescent species that are detected photometrically. Selectivities for phosphorus compounds and sulphur compounds are generally achieved by using a flame shield and specific optical filters to limit the background and hydrocarbon interferences. Unfortunately, the detector response to hydrocarbons is not completely blocked because of overlaps in the emission wavelengths for the various excited species, and thus, hydrocarbon compounds can still interfere with the analyte of interest.

The pulsed flame-photometric detector (PFPD) provides increased selectivity over the FPD by adding the domain of time to the analysis. In the PFPD, the pulsed-flame chemistry generates the chemiluminescent species in a time-dependent manner, and therefore, a time gate in the detector signal-processing firmware can be set to focus on the target emission. This feature of the PFPD provides for greater selectivity than for the conventional FPD, but the emissions for the various excited species still exhibit some overlap in time, and therefore, some interferences can still occur. By setting multiple time gates and after a simple multiple-compound calibration, the emission overlaps can be removed in real time during an analysis to provide enhanced selectivity for phosphorus and/or sulphur compounds. This enhanced-selectivity capability has been incorporated into the FM-2000PFPD manufactured by the CMS Field Products Group of OI Analytical. The FM-2000PFPD is a miniature, automated gas chromatograph, equipped with a PFPD.

### On-line headspace GC-monitor for measuring toxic VOC in food, soils and water at ppb levels

*Felix Behm, Airmotec AG 8308 Illnau, Switzerland*

The widespread use of plastics for food packing enables solvents, fuel and other toxic VOC to penetrate into food, beverages and drinking water. In reverse, flavour may evaporate through the walls of the packing. To fulfil the needs of quality control and safety requirements, those goods have to be under control for toxic contamination or loss of quality. With the new automated on-line head-space GC, up to 64 VOCs compounds can be traced automatically over hours, days or even weeks to measure the mass transfer rates through the package wall.

Examples have shown that gasoline and solvents are present in any goods stored in plastic containers, boxes, etc. The calculated transfer rates are high, so even short exposure to, e.g. gasoline vapours inside cars, results in high concentration of benzene, toluene and other organic compounds. High temperatures in summer can raise the concentration to dramatic highs.

Also, ingredients can drag in unwanted VOC. It has been shown that $CO_2$, used for sweet beverages, could bring in up to several hundreds ppb of benzene depending on the source of the $CO_2$.

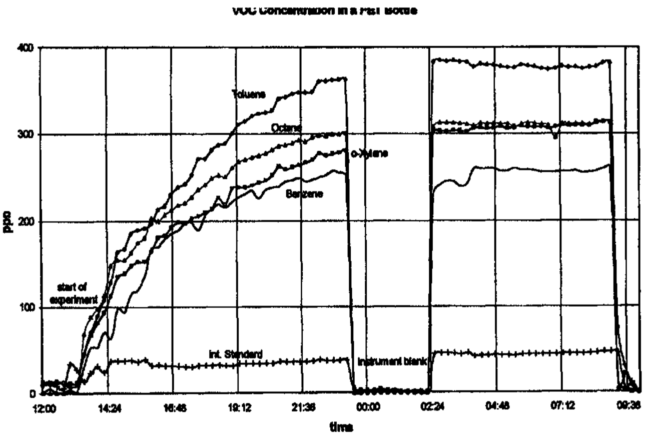

*Typical concentration plot versus time for some compounds in a 1.5 l PET bottle of drinking water. The bottle is exposed to benzene, toluene,* n*-octane and xylene vapours of 10 ppm at 20°C. Depending on their contribution factors, the different compounds show individual behaviour.*

### Performance of a 'universal' trace gas analyser designed for quality control in multiple gas cylinder-filling plants

*David Burges, Andrew Ditchman and Martin Smith, Unicam Chromatography, PO Box 205, York Street, Cambridge CB1 2SS, UK*

The trace analysis of high-purity industrial gases by gas chromatography involves complex multi-dimensional column switching in order to separate the impurities at parts per billion concentrations from the large column overload produced by the matrix gas. Generally, different column configurations and operating parameters are required for analysing each high-purity gas, mandating separate dedicated analysers for plants handling or filling a variety of gases.

The performance of a 'universal' trace gas analyser will be described, which employs the pulsed discharge ionization detector with a new multiple column configuration, allowing both backflushing and heart-cutting techniques to be accessed for any matrix gas. Software-selected method programs provide detailed trace analysis of the 'air' gases in a wide range of matrix gases, including hydrogen, helium, nitrogen, oxygen, argon, carbon dioxide and nitrous oxide, using the same hardware configuration. A sample loop purging system allows rapid changes of sample gas without long purging times.

Examples will be presented in this paper demonstrating detection limits in the low parts per billion range in a number of different matrix gases.

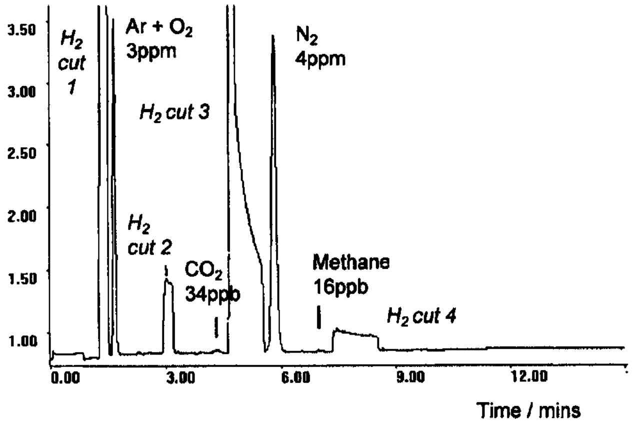

*Impurities in high purity hydrogen.*

### Enhanced performance of a micro-volume thermal conductivity detector for on-line gas analysis

*Teresa J. Lechner-Fish and Yang Xu, Daniel Industries, 9753 Pinelake Drive, Houston, TX 77055, USA*

The thermal conductivity detector (TCD) has been used extensively in on-line gas chromatography due to its simplicity, stability, durability and universal detection. Many analyses require the measurement of fixed gases and light hydrocarbons for which the TCD is ideally suited. The importance of the TCD in process gas chromatography is most evident when one considers the wide variety of analytes present in most gas streams.

Historically, three geometries have been used in TCD design: the flow-through, the semi-diffusion and the diffusion type cells. The flow-through cell has the advantage of faster detector response and more symmetric peaks. Using a pseudo-type cell design, these improvements can be achieved while retaining lower detection limits and broad dynamic range of the diffusion-type cells.

Although both filament and thermistor-type TCDS have been widely used in process gas chromatography, thermistor detectors have the advantage of being relatively inert to corrosive samples or samples containing trace amounts of oxygen. Being less sensitive to carrier flow perturbations than filaments, thermistors are better suited for the flow-through cell.

In this paper, the operating characteristics and performance of a 15-μl thermistor-type TCD will be discussed. In addition, repeatability data for a typical natural gas application will be presented.

### Analysis of haloacetic acids in water by a novel technique: simultaneous extraction–derivatization

*David Benanou, Francoise Acobas and J. L. Guinamant, Anjou Recherche, Analytical Research Department, 1 Place de Turenne, 'Immeuble le Duffy', 94417 Saint-Maurice Cedex, France*

A simple and novel method has been developed for the analysis of haloacetic acids (HAAs), disinfection by-products (DBPs) formed during water chlorination. This method can be used to determine six of these acids, including chloro, bromo and chloro/bromo acids. The validity and reliability of the method were tested over 6 months on 20 French water samples. Fifty millilitres of water is percolated over an ion-exchange resin, the strong acids are trapped and then eluted and simultaneously esterified to give their methyl derivatives by a methyl alcohol solution acidified with sulphuric acid. The esters are extracted by cyclohexane and analysed by gas chromatography with electron-capture detection (GC/ECD). Dalapon is used as a tracer in order to monitor the extraction process and trichloropro-

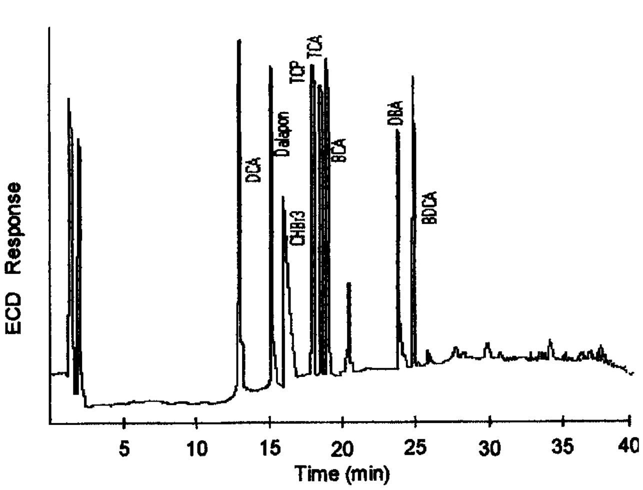

*Gas chromatogram of a real municipal water.*

pane as the internal standard. The detection limits for the HAAs analysed are between 0.1 μg/l and 0.2 μg/l. The analysis of different water samples showed that all the haloacetic acids, except monobromoacetic acid, were present.

Whatever the type of process used, it seems that the HAAs are clearly formed at the chlorination step, but it is notable that the untreated water, mainly for plant no. 1, already contained TCA. This molecule, whose presence was confirmed by GC/MS/NCl-$NH_3$, is in fact used as a herbicide in the form of sodium trichloroacetate. Because of its high volubility, it is thus possible to find TCA in surface water at certain times of the year. The total HAAs in water originating from surface waters were less than 50 μg/l, but could exceed 100 μg/l for water originating from reservoir waters. In underground waters, the concentration of HAAs was negligible.

### Instrumentation and methodology of continuous supercritical fluid extraction of water matrices

*Jiří Vejrosta and Pavel Karásek, Institute of Analytical Chemistry, Academy of Sciences of the Czech Republic, Veveří 97, 611 42 Brno, Czech Republic*

Direct supercritical fluid extraction (SFE) of water matrices is not as commonly used as solid samples extraction. The low water volubility (~1% mol) in supercritical or subcritical $CO_2$ permits direct extraction of various solutes from water-based matrices. The continuous mode of extraction possesses several advantages with respect to the static one. Among these, unlimited sample volume and faster phase equilibration are the most important ones. The presence of water increases the polarity of the supercritical phase.

The device uses a column packed with stainless steel coils (∅ 3 mm), into which the streams of water sample and $CO_2$ are delivered by two reciprocating pumps. The device is fully automated and can work in four regimes: co- and counter-current, and in both cases with or without water sample recycling.

The measurement of solute distribution coefficients and their use for analytical purposes will be discussed. The results, obtained by extraction of alcoholic beverages, will be presented.

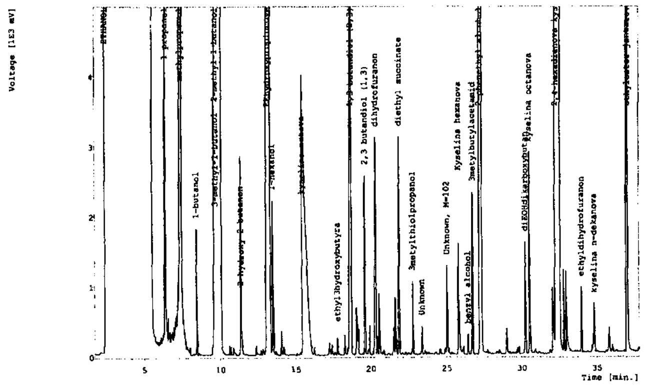

*Gas chromatogram of red wine extract obtained by direct SFE at 50 °C and 20 MPa.*

### Monitoring of environmental parameters with sequential injection analysis

*A. Cerdà and V. Cerdà, Department of Chemistry, University of the Balearic Islands, Carretera de Valldemossa km 7.5, E-07071 Palma de Mallorca, Spain*

Sequential injection (SI) was introduced in 1990 by Ruzicka and Marshall as a novel development in the field of continuous analysis. This approach offers improved features over flow injection (FI) with respect to the optimization procedures involved, reagent consumption and flexibility. Together with versatile software, it provides a powerful tool for environmental applications, as a single configuration can be adapted to different analytical pathways and used for multicomponent analysis and/or multidetection applications.

An overview of the potential of SI in environmental analysis is herein presented. To this end, several approaches to the determination of key parameters, e.g. nitrogen ($NO_3^-$, $NO_2^-$, ammoniacal and total nitrogen), phosphorous ($PO_4^{3-}$ and total phosphorous), iron (with and without preconcentration), $Ca^{2+}$, $Mg^{2+}$, $Cl^-$ and pH are briefly described. Furthermore, the recent application of SI to the design of a water quality monitoring system for on-line near real-time measurement of BOD, COD, TSS, detergents, and nitrogen and phosphorous compounds is also reported. The system has been successfully used for automated analysis of the target compounds in waste waters, with and without (Pastel UV) chemical reactions.

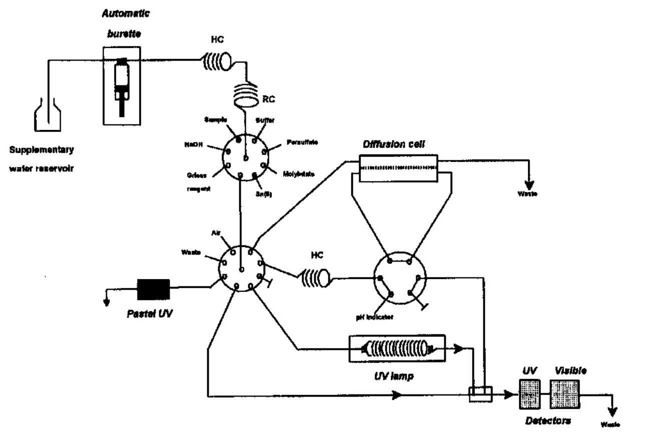

*Sequential injection water quality monitoring device based on spectrophotometric measurements.*

### On-line SPE-PTV-GC-MS: a vision to the determination of micro-pollutants in surface water

*Sjaak De Koning, Wil Van Egmond, Hans-Gerd Janssen[1] and Bert Ooms[2], Atas International B.V., P.O. Box 17, 5500 AA, Veldhoven, The Netherlands; [1]Eindhoven University of Technology, P.O. Box 513, 5600 MB, Eindhoven, The Netherlands; [2]Spark Holland B.V., P.O. Box 388, 7800 AJ, Emmen, The Netherlands*

The monitoring of water samples for the presence of organic pollutants at trace levels requires sensitive and selective methods. Because surface water legislation is becoming more stringent every year, new methods have to be developed. To meet the sensitivity and selectivity demands, the determination of organic substances in water commonly involves selective isolation of the compounds of interest, separation by means of a chromatographic technique and detection using a sensitive and selective detector.

The present study was intended to demonstrate the possibility of fully automated SPE-PTV-GC-MS. First the PTV-GC-MS procedure was optimized, followed by the optimization of the SPE procedure. Finally, both procedures were hyphenated.

The performance of the fully automated system was tested by analysis of triazines. RSD values were determined by spiking surface water from the River Meuse (Eijsden, The Netherlands) at the 1 μg/l level and were found to be in the range of 5%. The retention time RSDs are in the 0.05% range. For all compounds, the linearity was quite good and the limits of detection are in the range of ng/l.

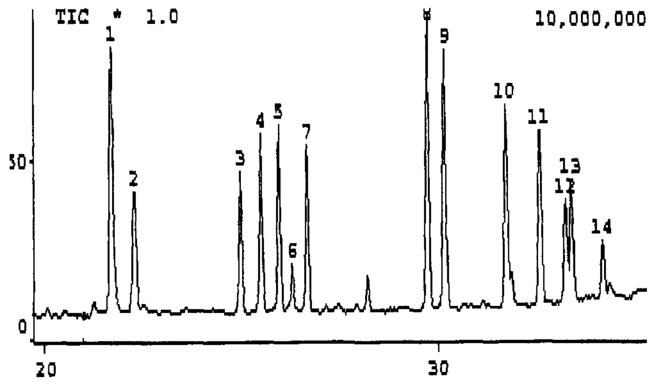

*Full scan (40–350 amu) GC–MS chromatogram of surface water spiked at the level of 1 μg/l. 1 = ethoprophos, 2 = desethyl atrazine, 3 = simazine, 4 = atrazine, 5 = propazine, 6 = N-butylbenzenesulphonamide, 7 = terbutylazine, 8 = co-elution of metribuzine and desmetryne, 9 = diisobutyl phthalate, 10 = prometryn, 11 = terbutryn, 12 = cyanazine, 13 = dibutyl phthalate, 14 = parathion ethyl.*

### Combination of flow injection technique and preconcentration of metals using organic completing reagents

*Vladimir V. Kuznetsov, Elena A. Filatova and Berta M. Shteingrat, Department of Analytical Chemistry, Mendeleyev University of Chemical Technology of Russia, 9 Miusskaja Sq., 125047 Moscow, Russia*

Flow injection technique is the best way to automate the procedures of wet chemical analysis. Nevertheless, the sensitivity of the analytical reactions used is not used completely because of dispersion phenomena. Different techniques of analytical preconcentration are in wide use in this method. Among the different techniques of preconcentration, coprecipitation is not usually a very attractive procedure. The new hyphenated way of high effective on-line filtration preconcentration–dissolution in spectrophotometric flow injection analysis has been developed.

The main chemical idea exists in usage of organic completing reagents' systems, which promote a poorly soluble in water precipitant containing the metal to be determined at the trace concentration level as coloured chelate. The concentrate is filtered through the on-line filter automatically and, further, is dissolved in the dose of organic solvent and a flow signal is registered. The simple flow system can be used for as-mentioned preconcentration–dissolution measurement of the absorption procedure.

The coprecipitation of elements using the organic chelating reagents is a powerful chemical tool for determination of metal in environmental chemistry. There is the opportunity to determine the different metals, including those which are toxic, radioactive at the trace concentration level. The different kinds of chemical coprecipitation systems for flow technique for determination of uranium, strontium, chromium(III) will be discussed in this paper.

### Microminiaturization: a clinical laboratory perspective

*Larry J. Kricka, Department of Pathology and Laboratory Medicine, University of Pennsylvania, Philadelphia, PA 19104, USA*

Microminiaturized analytical devices and systems represent one of the most active current areas of research and development in the analytical sciences. Factors important to this growth are the numerous initiatives in high-throughput massively-parallel drug discovery assay systems, the continued interest in developing clinical assays for the point-of-care (bedside, home), and a general goal of integration of multi-step analytical procedures onto a single small chip device ('lab-on-a-chip').

In drug discovery, assays are being formatted onto microwell plates with ever increasing numbers of wells with smaller and smaller volumes (e.g. 20 000 wells; 50 nl) or onto glass microchips capable of parallel testing of microvolumes of sample. Microminiaturized assays for the clinical laboratory are based on various types of chip devices [e.g. 'DNA chips', 'genechips', 'genosensors', 'bioelectronic chips', and 'microdot' (antibody) chips] constructed on 1-$cm^2$ pieces of silicon, glass or plastic.

A range of clinical assays and techniques have been adapted to the microchip format including nucleic acid assays (e.g. PCR, DOP-PCR, LCR, mutation analysis, sequencing), capillary electrophoresis, semen analysis, immunoassay. Particularly significant has been the degree of integration achieved in the microchip format (e.g. microfluidic control, thermal control, sample preparation, analytical reaction, detection of products).

The clinical laboratory and the point-of-care environment have specialized requirements and restraints. Microchips offer a number of potential benefits but there are still unresolved issues concerning the user interface, cost effectiveness, the degree of miniaturization that is desirable in view of the very low concentrations of some analytes in blood (e.g. hormones, e.g. thyroid-stimulating hormone), and the menu of tests most appropriate for a microchip format. The future prospects and opportunities for microminiature analysis using microchip-based devices in these contrasting environments will be examined, and possible barriers to their widespread utilization discussed.

### US EPA's environmental technology verification program

*Penelope Hansen, US Environmental Protection Agency, National Risk Management Research Laboratory, Environmental Technology Verification Program Office, 401 M St. SW, Washington, DC 20460, USA*

This presentation on EPA's environmental technology verification program (ETV) will include benefits of verification; the ETV goals; important operating principles for the ETV program; a description of the 12 ETV pilots; explanation of the ETV verification process; the importance of an ETV verification statement; outputs to-date including verified technologies and other testing activities; a preliminary analysis of the ETV markets; and quality criteria for a verification program.

The ETV program is a service of the EPA designed to accelerate the development and commercialization of improved environmental technology through third party verification and reporting of performance. The goal of ETV is to verify the performance characteristics of commercial-ready environmental technologies through the evaluation of objective and quality-assured data so that potential purchasers and permitters are provided with an independent and credible assessment of the technology that they are buying or permitting. ETV is intended to expand the environmental technology choices of public and private decision-makers, both in our country and abroad.

ETV is a voluntary program that seeks to make objective performance information available to all of the actors in the environmental marketplace for their consideration and to assist them in making informed technology decisions. ETV does not rank technologies or compare their performance, label or list technologies as acceptable or unacceptable, or seek to determine 'best available technology', or approve or disapprove technologies. The program does not evaluate technologies at the bench- or pilot-scale, and does not conduct or support research.

The program now operates 12 pilots covering a broad range of environmental areas. ETV has begun with a 5-year pilot phase (1995–2000) to test a wide range of partner and procedural alternatives in various pilot areas, as well as the true market demand for and response to such a program. In these pilots, EPA utilizes the expertise of partner 'verification organizations' to design efficient processes for conducting performance tests of innovative technologies. EPA has selected its partners from both the public and private sectors including federal laboratories, states, industry consortia and private sector facilities. Verification organizations oversee and report verification activities based on testing and quality assurance protocols developed with input from all major stakeholder/customer groups associated with the technology area.

### On-line multi-element determination with laser-induced breakdown spectroscopy

*Reinhard Noll, Lazlo Peter, Volker Sturm, Ingo Mönch, Risto Hakala*[1]*, Jorma Viirret*[1]*, Fraunhofer-Institut für Lasertechnik (ILT), Steinbachstraße 15, D-52074 Aachen, Germany;* [1]*Rautaruukki Steel, Technical Services, P.O. Box 93, FIN-92101 Raahe, Finland*

Laser-induced breakdown spectroscopy (LIBS) enables a contactless material analysis over distances in the range of some 10 cm, and thus a simplified access to the measuring object in a production line. However, the analytical performance of LIBS in terms of limits of detection has to be improved considerably to fulfil typical process requirements. The objectives of current R&D activities at ILT are the optimization of both the excitation of the material plasma, and the time and spectrally resolved detection of the atomic emission.

The research plan is focused on iron-based materials, low- and high-alloyed steels. The interaction of modified Q-switched pulses of a Nd:YAG-laser with the metallic specimens was investigated. The atomic emission is coupled to a Paschen–Runge spectrometer covering a spectral range from 120 nm to 400 nm with a spectral resolution of 20 pm. The photomultiplier signal lines are linked to fast gateable integrators.

The limit of detection ($LOD_{3\sigma}$) for carbon in low-alloyed steel was improved to 12 ppm. For the first time, sulphur and phosphorus were quantitatively determined with a similar LOD. Further efforts aim to push the LOD below the 10 ppm level.

Another application of LIBS is the on-line identification of high-grade steel qualities. An automated inspection machine was set up and integrated in a production line of a manufacturer of different types of stainless piping components. In the first months after the start of operation the machine has identified nearly 30 000 workplaces. With this application, the technical feasibility for an on-line multi-element determination with LIBS was demonstrated successfully.

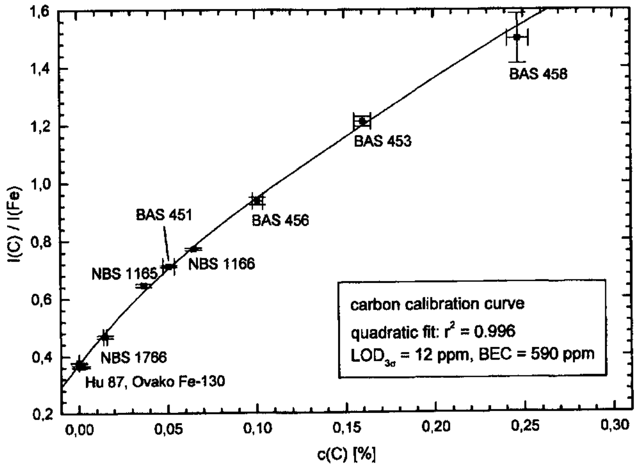

### Applications of a novel spectral curve-fitting technique in ICP-AES

*Chris Webb, Andrew T. Zander, Philip V. Wilson*[1]*, Gabriel Perlis*[1] *and Doug Shrader*[2]*, Ginzton Research Center, Varian Associates, 3075 Hansen Way, Palo Alto, CA 94304, USA;* [1]*Varian Australia, Optical Spectroscopy Instruments, 679 Springvale Road, Mulgrave, Victoria 3170, Australia;* [2]*Varian Associates, 201 Hansen Court, Suite 108, Wood Dale, IL 60191, USA*

A novel fast automated curve-fit technique (FACT) has been designed to provide on-line spectral peak-fitting in inductively-coupled plasma-atomic emission spectrometry (ICP-AES). This new algorithm will operate in situations of varying complexity, and is able to compensate accurately for any wavelength drift that may occur. The method uses argon calibration lines to ensure against drift problems. Model components are offset as necessary resulting in better than 0.1 pixel accuracy for relative location of models and data. In order to offset arbitrary peak shapes by fractional pixel amounts, each model component is decomposed into the sum of one or more Gaussians and the residuals. Fractional pixel offsets are then effected by shifting the Gauss functions combined with linear interpolation of the slowly changing residuals.

The technique can be used to evaluate accurately even strongly overlapped peaks, and is also of particular value in situations where it can be used to effectively extract small peaks from complex backgrounds with greater accuracy than conventional background correction methods. Additionally, it can be used to eliminate flicker noise by simultaneous background correction resulting in improved detection limits.

In this paper we will discuss applications of FACT to a variety of ICP problems, and will demonstrate its accuracy and precision in relation to Cd/Fe analyte/interference line pairs near both 214 and 226 nm. The method has also been applied in a case—Se/Fe—where spectral overlap was not as severe, but where peak-fitting works better than conventional background correction.

### An intelligent semi-quantitative data processing method for ICP-AES using spectral pattern recognition

*Mark R. Anderson, Andrew T. Zander, Philip V. Wilson*[1] *and Stewart G. Hamilton*[1]*, Ginzton Research Center, Varian Associates, 3075 Hansen Way, Palo Alto, CA 94304, USA;* [1]*Varian Australia, Optical Spectroscopy Instruments, 679 Springvale Road, Mulgrave, Victoria 3170, Australia*

Innovations in the design of CCD detectors for inductively-coupled plasma-atomic emission spectroscopy (ICP-AES) have dramatically increased wavelength coverage. The new VistaChip detector contains 70 000 pixels and covers the range from 167 to 785 nm. As a result, the number of available elemental emission lines from the sample is greatly increased and automated analysis techniques are needed to make the most efficient use of this amount of information. This presentation will describe software designed to take advantage of the VistaChip wavelength coverage to automatically detect all elements present in the sample with a minimum of *a priori* knowledge. The software also generates a list of intelligently selected analysis lines for method development.

General detection methods were developed using neural networks to identify the presence of emission lines in the data and to recognize element-specific patterns of line intensity. The elemental intensity patterns are determined using a set of 10 'index' lines for each element. The index lines are selected on the basis of measured detection limits for each element on the Vista instrument. The probability of element presence is estimated from the combination of the presence of the index lines in the sample data and the similarity between the sample pattern and a reference intensity pattern. The reference intensity patterns were also established by measurement on the instrument. Concentration ranges for detected elements are estimated by comparison to the reference intensity data for the index lines. The index lines are qualified for method development during the estimation of the concentration range, and lines which either fail the peak presence algorithm or whose intensity falls outside the estimated concentration range are rejected.

The software operates as a 'one-button' solution for non-experts to generate a table of suspected elements, semi-quantitative estimates of their concentrations, and a list of useable lines as a basis for method development. Identification and semi-quantitative determination is rapid (<20 s) even for a large number of elements (>40). Further details of the software operation will be discussed and results of a variety of sample analyses presented.

### Automated headspace analysers designed to ensure the highest confidence of results in the pharmaceutical and forensic field

*Fausto Pigozzo, Don E. Clay and Rollen Anderson, ThermoQuest Corporation, 2215 Grand Avenue Parkway, Austin, TX 78728, USA*

Determination of volatile toxic compounds in biological fluids and tissues is currently accomplished with approved methodologies that include instrument verification performance and quality check between the real samples. These procedures are very effective from one side to avoid any interference from the sample matrix and ensure an accurate measurement, but they increase enormously the manual workload required to characterize a sample. This paper illustrates the performance of dedicated analysers designed to automate to the highest possible extent the determination of volatile organic compounds.

Static headspace is used as the sampling technique because of its capability to provide accurate determination of volatiles in complex matrices eliminating most of the sample preparation steps. Simultaneous, dual-column confirmation analysis provides confidence of results. Additionally, a software package capable of controlling injection sequences with programmable automated actions defined upon the results of QC check, standard or duplicates analysis, completes the analysers. The advantages of the analyser, in term of costs saving, are discussed with respect to blood alcohol analysis and analysis of organic impurities in pharmaceutical bulks. Results are shown where speed of analysis and accuracy of results are both improved, together with a reduction in the amount of manual sample handling.

The automated overlapping mode used by the headspace autosampler to condition the samples, the fast cooling performance of the GC oven, the single automated injection into two dissimilar megabore columns, all together enhance the performance of analyses in a shorter time while providing the confidence of results.

The sensitivity achieved easily surpasses the required detection limits of the methods. The experimental data illustrate the precision and accuracy of the results provided.

### Characterization and application of a flow cell for on-line electrochemistry/mass spectrometry in non-aqueous solvents

*Zhang Tianyi and Brajter-Toth Anna, Department of Chemistry, University of Florida, Gainesville, FL 32611, USA*

In previous work (Regino and Brajter-Toth, *Anal. Chem.*, **69** (1997), 5067–5072), an electrochemical cell for on-line EC/MS has been described. The cell has the advantages of adjustable volume, easy access to the working electrode for resurfacing or replacement, and the capability to withstand high back pressure. In this work, the characterization and the application of the cell to on-line EC/MS in non-aqueous media will be discussed.

The cell was characterized with 1.0 mM $K_3Fe(CN)_6$–0.1 M $NH_4OAC$ aqueous solution first before being used in non-aqueous media. The results are consistent with those described before. A steady-state thin-layer behaviour is observed with cyclic voltammetry with scan rates below 10 mV/s at a flow rate of 1.0 ml/min. The calculated thin-layer cell thickness is 50.8 µm. The cell conversion efficiency is affected by the flow rate. As the flow rate increases, the residence time of analyte in the cell becomes shorter, and the fraction of the analyte introduced into the cell that undergoes electrochemical reaction becomes lower. As a result, the cell conversion efficiencies determined with ferricyanide range from 2.21 to 0.48% at flow rates from 0.25 to 2.0 ml/min.

To characterize the cell in non-aqueous solvents, a test analyte, triphenylamine (TPA), was chosen. TPA undergoes a one-electron transfer oxidation in acetonitrile to produce a cation radical. The cation radical then undergoes a para–para coupling reaction to form tetraphenylbenzidine (TPB). TPB can be oxidized in two one-electron transfer steps to generate a dication intermediate. TPA electro-oxidation does not generate side products. In our experiment, the electrochemical oxidation of TPA in acetonitrile and DMSO solvents on Pt and rough pyrolytic graphite (RPG) electrodes, with tetrabutylammonium perchlorate (TBAP) and tetraethylammonium perchlorate (TEAP) as electrolytes, has been evaluated. The best signal-to-noise response is achieved with TBAP as electrolyte and acetonitrile as solvent. The off-line flow injection analysis (FIA) was performed prior to the on-line EC/MS experiments. With the potential of RPG working electrode controlled at +1800 mV versus Pd wire, 1.0 mM TPA–0.1 M TBAP mixture was injected into the mobile phase of 0.1 M TBAP and acetonitrile. The hydrodynamic voltammograms constructed by FIA are in agreement with off-line cyclic voltammetry. A linear current response is observed for TPA at concentrations as low as 20 µM. A range of cell conversion efficiencies from 17.55 to 4.32% was determined from FIA as a function of flow rate and injection volume.

In on-line EC/MS experiments, the electrolyte and solvent have to be optimized to facilitate both vaporization and ionization of the analyte. Although TBAP is not volatile, the mass ion peaks from TPA can be easily seen with particle beam LC interface in the presence of 1.0 mM TPA–0.01 M TBAP mixture in acetonitrile. The preliminary results will be discussed.

### Determination of inorganic mercury by the use of immobilized mercuric reductase and flow injection analysis

*R. Wayne Ross, Salam Abdo and Stuart J. Chalk, Department of Natural Sciences, University of North Florida, Jacksonville, FL 32224, USA*

The determination of mercury using cold-vapour atomic absorption (AAS) and atomic fluorescence spectrometry (AFS) has proved to be the method of choice in the 1990s. The integration of flow injection analysis sample processing with AAS and AFS has produced detection limits down to parts per trillion levels with gold gauze preconcentration. However, the methodology employed to-date requires large amounts of reductant [borohydride or tin(II)], acid (nitric or sulphuric) and purge gas (argon). This generates a large amount of waste.

In an effort to adapt cold-vapour technology to remote analytical instrumentation, we have developed an immobilized mercuric reductase reactor for conversion of Hg(II) to Hg(0). This enzyme specifically catalyses mercury reduction in the presence of two co-factors; NADPH and a sulphydryl compound (e.g. β-mercaptoethanol). Immobilization of the enzyme of Sepharose beads does not decrease the activity of the mercuric reductase and makes it a viable alternative for conversion of Hg(II). Production of mercury vapour is also on a similar time-frame to the conventional methodology and generates much less waste.

This paper will present details of the production of the enzyme, immobilization of the enzyme on inert supports, and characterization of the enzyme reactor in terms of activity and stability. The immobilized enzyme reactor is integrated into a Perkin Elmer FIMS-100. Results of the analysis of Hg(II) contaminated water and a discussion of interferences will also be presented in this paper.

### Sub part per trillion levels of mercury without amalgamation using a new atomic fluorescence spectrometer

*W. T. Corns and P. B. Stockwell, PS Analytical, Arthur House, Crayfields Industrial Estate, Main Road, Orpington, Kent BR5 3HP, UK*

In 1987, the world's first fully automated mercury analyser, based on atomic fluorescence (AFS), was introduced. This inherently linear technique offers advantages over alternative approaches based on atomic absorption and, whereas previously introduced systems for trace elemental analysis using AFS floundered, the combination with a vapour generation stage to evolve the mercury vapour allows this approach to flourish.

A new instrument, the Millennium Merlin system, which combines both the vapour generation stage and the AFS detector system, will be discussed. By careful attention to detail and new developments, this instrument further extends the capabilities of the approach for mercury analysis.

Performance data will be presented illustrating the ability of the atomic fluorescence process to achieve method detection levels in the region of 0.1 ppt routinely. Another valuable feature of the instrumentation is its wide linear dynamic range over seven orders of magnitude, and the value of this to environmental analysts will be demonstrated in this paper.

### Toward a miniature Hg analyser

*J. T. Sharples, G. Mew, A. Nathan and V. Karanassios, Guelph-Waterloo Center for Graduate Work in Chemistry, University of Waterloo, Department of Chemistry, Waterloo, Ontario, Canada N2L 3G1*

The determination of Hg in gaseous, liquid or solid samples has been receiving continued attention due to potential health hazards. In terms of analytical methods and, in most instances, Hg-contaminated samples must be brought to the laboratory. There are many instances, however, in which it would be desirable (or even necessary) to bring the laboratory to the sample rather than the sample to the laboratory. In such cases, a portable Hg analyser is required.

We have been working toward this conceptual goal by developing a miniature atomic fluorescence cell (AFS) using wet chemical etching technology borrowed from the semiconductor industry. The AFS microcell has been tested with gaseous microsamples using an injection port as a sample introduction system. Liquid microsamples and powdered solids (as slurries) were analysed by placing microlitre volumes into the metal-cup of an in-torch vaporization (ITV) sample introduction system (which was originally developed in my laboratory for inductively coupled plasma (ICP) spectrometry).

In this paper, fabrication technology will be briefly discussed, results obtained using gaseous, liquid and solid microsamples will be described in detail, and future research directions will be outlined.

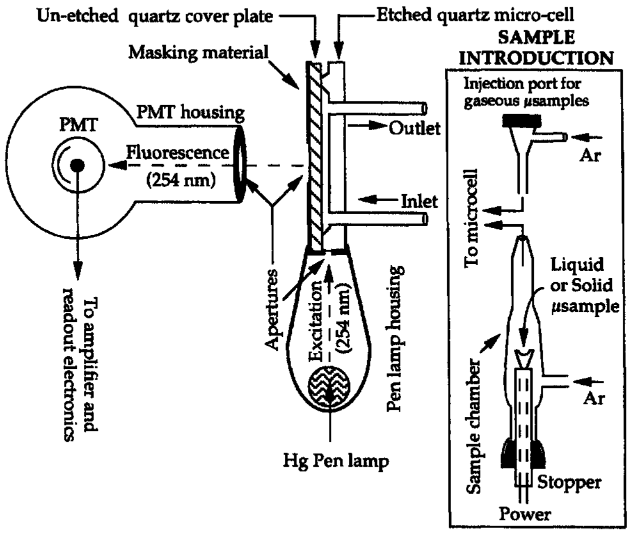

*Experimental set-up (illustration not to scale).*

### On-line analysis of effluent streams for mercury in the waste incineration industry

*W. T. Corns, P. B. Stockwell and N. K. Brahma, PS Analytical, Arthur House, Crayfields Industrial Estate, Main Road, Orpington, Kent BR5 3HP, UK*

In the waste incineration industry, monitoring mercury levels and its removal prior to transfer to waste is a significant problem. Controlling and monitoring the analyte to reduce mercury levels poses distinctive challenges for the analytical chemist. The complexity of the chemistry prior to and after the removal of mercury cannot be underestimated.

The authors have developed an important advance in methodology by applying discrete sample injection techniques to analyse samples, e.g. 50% caustic soda on a repetitive basis. Previously, this approach had shown useful applications for the analysis of mercury in 98% sulphuric acid. Such an approach offers advantages over conventional on-line measurements as very small samples are introduced to the instrumentation, thus avoiding matrix interference effects. The atomic fluorescence detection system, with its low detection levels and wide linear dynamic range, allows this procedure to be a viable option in the chloralkali industry. Instrumental systems have been developed for the analysis of mercury in 50% caustic streams and associated effluent streams. Recently, this technology has been transferred to the waste incineration industry.

The challenges provided by these measurements and the procedures developed to analyse the mercury levels routinely and automatically will be described in this paper. Results will be presented from an installed monitoring system.

### Direct AA mercury determination in the samples with complex matrix

*Sergey E. Sholupov, Sergey E. Pogarev, Vladimir V. Ryzhov and Alexander Ganeev, Earth Crust Research Institute, St. Petersburg State University, Universitetskaya nab., 7/9, St. Petersburg, 199034, Russia*

Direct atomic absorption mercury determination (without any pretreatment procedures) in the samples with complex matrix (foods, vegetation, oil and its products, soils, sediments, various biological materials) is complicated by presence of organic compounds. When such samples are atomized, much smoke is produced and practically in all cases the total absorption in the cell is out of the working range of the spectrometer. Using a spectrometer with background correction—Zeeman atomic absorption mercury spectrometer RA-915 and a developed two-chamber catalyst atomizer—the direct mercury determination problem is solved.

After inserting the sample in the first chamber of the atomizer, it is heated up to 800 °C (all mercury compounds are known to be dissociated at such temperatures). Less volatile mercury compounds are dissociated, but more volatile species, e.g. mercury organic compounds, can leave the first chamber as compounds during warming up of the chamber. In addition, presented organic compounds of the matrix originate much smoke and variously compounds which are able to produce strong background absorption in the analytical cell. All products are transported from the first chamber to the second one by the carried gas (in our case it is air). The second chamber is continuously heated at ~800 °C. There, all mercury compounds are dissociated, and smoke and interference compounds are burst producing mostly carbon dioxide and water. The rest background absorption is eliminated by Zeeman corrector of the spectrometer. The catalyst improves the efficiency of oxidation and dissociation in the second chamber.

### A fully automated hydride generation device for inductively coupled plasma spectroscopy

*Yutaka Hayashibe and Minoru Takeya, Materials Characterization Center, Central Research Institute, Mitsubishi Materials Corporation, 1-297 Kitabukuro-cho, Omiya, Saitama, 330-0835, Japan*

Current techniques and devices available for the determination of hydride-forming elements by inductively coupled plasma-atomic emission spectroscopy (ICP-AES) are limited by the requirement for large sample volumes. Segmented flow techniques that allow the use of smaller volumes can cause problems with the stability of the plasma discharge due to the introduction of hydrogen gas during analysis. Ideally, a hydride generation device should match the capability of the simultaneous ICP spectrometer system in allowing rapid, simultaneous determinations using a minimum amount of sample and reagents. Also, where different elements require different reduction conditions, the changeover from one reagent and its concentration to another should be automated, rapid, requiring little additional sample and have no effect on the stability of the plasma discharge.

We have developed a fully automated hydride generation device that allows either the simultaneous or rapid sequential determination of seven hydride-forming elements. The proposed hydride generation system featuring a newly designed pump for varying reagent concentrations and automated switching valve will be discussed. The optimum system parameters for multi-element determination of hydride-forming elements will also be discussed. With this system, we have achieved enhanced sensitivity and selectivity for the determination of these elements in materials used for research and product development at our company.

### Protein identification and characterization using MALDI-MS and ESI-MS/MS on a quadruple time of flight hybrid tandem mass spectrometer

*Mark McDowall, Robert Bordoli, Jeffery Brown, Anthony Gilbert, John Hoyes and James Langridge, Micromass UK, Floats Road, Wythenshawe, Manchester M239LZ, UK*

Complete genome sequences are already available for some small organisms (yeasts, bacteria and nematode worms) and within the next decade the sequence of the human genome will have been completed. However, understanding the mechanisms of cellular processes will require the elucidation of detailed protein maps from high-throughput proteomic studies.

Separation of proteins from cell lysates or sub-cellular domains by two-dimensional polyacrylamide gel electrophoresis (PAGE) has been a useful way of visualizing these complex systems. MS has proved to be a powerful way of characterizing proteins from cell lysates. MALDI is of great benefit in these studies as it may be configured for automated sample analysis and data processing. In our approach to maximize MALDI throughput, post-acquisition data processing automatically filters the mass spectra into lists of mono-isotopic masses, which are then sent to a client-server database for protein identification. Proteins which are not identified by this method, or which present ambiguous results, can be reanalysed by ES MS/MS to give sequence information for some of the individual peptides in the digest mixture. This may be submitted to the client-server database for protein identification. This sequence information may be used to search the protein and genome databases for protein identification, and has the added advantage that EST databases, which are growing at a faster rate, may be searched. In the event that the protein remains unidentified at this stage, the sequence information may be used to build oligonucleotide probes for conventional cloning.

When *de novo* sequencing of the MS/MS data is required, the high resolution and mass accuracy provided by a Quadrupole-TOF instrument is an advantage. For particularly complicated problems, digestion in 50% enriched $^{18}$O-labelled water to attach a characteristic isotopic tag to the C-terminal ends of the digest fragments may be used to good effect. Examples of this strategy will be shown using a Q-Tof$^{TM}$ (Micromass, Manchester, UK) ESI-MS/MS instrument.

### Solid-phase extraction/MALDI-MS for on-probe sample clean-up of biological samples

*Ron Orlando, Maria E. Warren, Adam H. Brockman and Li Zhang, Complex Carbohydrate Research Center and the Departments of Biochemistry & Molecular Biology, and Chemistry, University of Georgia, 220 Riverbend Road, Athens, GA 30602, USA*

Biological samples typically contain inorganic salts, buffers, chaotropic agents, preservatives, detergents and various other solubilizing agents. Biologists employ these compounds in preparing samples for several reasons, including maintenance of a non-toxic environment for cells, stabilization of solvated samples, and maintenance of enzymatic or other biological activity. In addition, many separation strategies used in the isolation of biological molecules require high concentrations of these agents. For instance, carbohydrates can elute from a high-pressure anion-exchange column in over 1 M sodium hydroxide/sodium acetate. However, these contaminants cause problems for subsequent characterization, including mass spectrometry.

We have developed an on-probe sample clean-up strategy based upon chemically modifying the surface of a MALDI probe so that it is capable of extracting biological polymers from the solution while having little or no affinity for the contaminants in the sample. We have called this approach SPE/MALDI-MS, as the probe derivatizations we use provide surfaces that are chemically similar to the media used in solid-phase extraction (SPE). For example, we have created MALDI probes with a hydrophobic self-assembled monolayer (SAM), a surface chemically similar to the C18 stationary phase commonly employed in SPE. These hydrophobic SPE/MALDI-MS probes are capable of binding peptides/proteins via hydrophobic interactions, and allow both inorganic salts and chaotropic agents to be removed with aqueous washes. A major limitation in hydrophobic SPE/MALDI-MS probes is the low degree of 'wetability' of the surface. The hydrophobic nature of the surface combined with the aqueous solutions of the sample result in strong sample surface tension and the formation of a near-perfect sphere on the surface, all of which severely limits the amount of peptide that can be extracted.

The inherent limitation of the hydrophobic surfaces provided the impetus for the development of probes with 'wetable' surfaces for SPE/MALDI-MS. Here, we describe the use of MALDI probes possessing ionic functional groups. MALDI probes modified in this manner have excellent 'wetability' character, and can rapidly extract peptides/proteins via ionic interactions from $\leq$1-ml volumes of sample solutions placed directly on the modified probe. We have found that ion-pairing SPE/MALDI probes are a practical solution for analysing very small volumes of peptide/protein solutions contaminated with high levels of inorganic salts, buffers, detergents, chaotropic agents and other solubilizing agents. Similarly, we have been able to analyse oligonucleotides and oligosaccharides from solutions saturated with sodium chloride.

### NIR calibration method from a remote location and during on-line operation

*Greg Lankford and Irena Zilberman, Petrometrix, 1211 Richmond Ave., Suite #B109, Houston, TX 77082, USA*

The methodology of calibrating the NIR (near-infrared) analyser is an important step for quick analyser implementation and for maintaining the analyser up-time, during normal operation, to as close to 100%, as possible.

Two different distinct situations are noted. In the simple situation, the customer intends to install the analyser on active process streams. The way to perform calibration in this case is that several months before the analyser installation, the customer collects samples and a few days before the installation, the samples are measured in the plant laboratory. A reasonable calibration model can be developed in the lab unit and then later transferred to the on-line system.

In the more complex case, the plant intends to install the analyser on a newly constructed processing unit. In this situation, samples are not available prior to the processing unit start-up and in addition, the analyser is needed in the start-up phase. Over the last decade, Petrometrix has established a huge database containing thousands of samples from all over the word characterized by numerous properties, in order to deal with this type of situation. A special technique was developed, which utilized only a number of samples from the new process. This technique was used in conjunction with the database to track the process variation immediately after start-up. Model fine-tuning is performed while the system tracks the process variation, with no down-time.

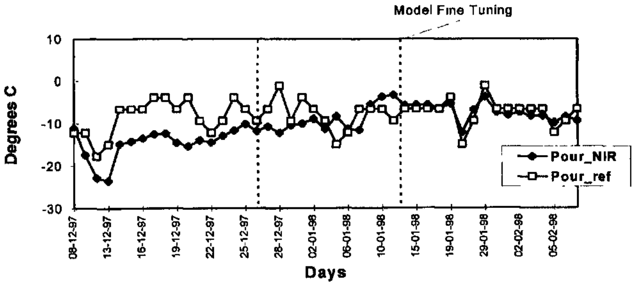

*Pour point of diesel fuel (on-line Dec. 1997–Feb. 1998).*

### Near-infrared spectroscopy in on-line analysis of diesel streams

*Ilan Sela, Greg Lankford and Irena Zilberman, Petrometrix, 1211 Richmond Ave., Suite # B109, Houston, TX 77082, USA*

On-line analysis of various properties of the refinery's diesel streams, e.g. distillation point, cloud point and pour point, are carried out using the NIR (near-infrared) spectroscopic technique.

A fast sample loop connects the intrinsically safe sample probes to the diesel streams. Two distillation towers are monitored using a single multiplexed NIR system, with an additional four probes installed on other streams in the same refinery.

The NIR instrument is of an innovative design, using standard telecommunication optical fibres to separate the sample probes. These are located next to the process stream of the analyser, which is located in the plant control room. The longest fibre optic cable being used in this installation is 2000 feet in length.

The effectiveness of the newly developed concept of a single analyser in the DCS room, multiplexed to various process streams, has proven to be a cost-effective and reliable method.

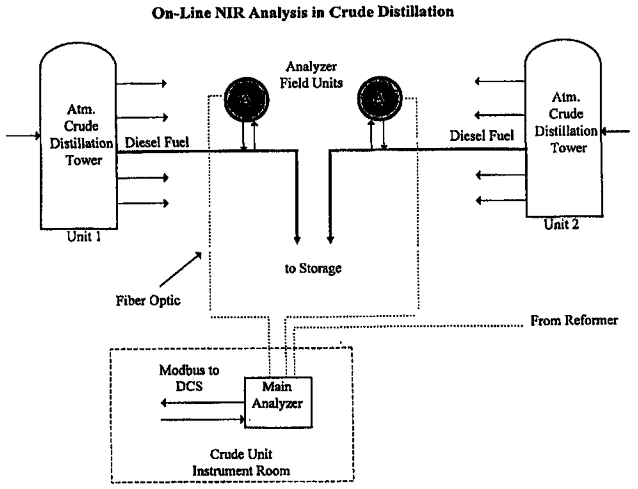

*Plant system layout.*

### Acid evaporation of digested samples for ICP-MS analysis using microwave heating in conjunction with a vacuum source

*Greg Leblanc and Leslie Rhodes, CEM Corporation, POB 200, Matthews, NC 28106-0200, USA*

The conventional sample preparation step for elemental analysis used to be a very simple, yet time-consuming, hot plate procedure. The objective was to produce a homogeneous, aqueous solution for the analyst. The analyst was even somewhat forgiving when there were residual organics left in the samples.

The analytical tools for elemental analysis have advanced significantly in the modern analytical laboratory. We have gone from atomic absorption to inductively coupled plasma-optical emission spectroscopy (ICP-OES) to the current standard, inductively coupled plasma-mass spectrometry (ICP-MS). The ICP-MS has detection limits in the ppt range with multi-element analysis capabilities in under 2 min per sample.

However, this increased analytical capability has not come without increased demands on the sample preparation process. Closed vessel microwave digestion has significantly improved the throughput capabilities to better match up with the analyser. This technology also provides very rigorous conditions to put materials into solution that could not be achieved with conventional hot plate methods.

But this is still not good enough. The analyst now wants their solutions for analysis to be free of any fluoride and chloride ions, and have the nitric acid concentration at levels less than 5%. This paper will present performance data on a new accessory for closed vessel microwave systems to allow vacuum-assisted acid evaporation of digestates. This step is performed under temperature feedback control without transfer of the sample to minimize contamination. Evaporation times are 30–35 min for up to 12 samples containing 15 ml of acid.

### A sample preparation expert-database system for microwave digestion

*Fei Qin, Jason Brown and H. M. 'Skip' Kingston, Duquesne University, Department of Chemistry and Biochemistry, 600 Forbes Ave., Pittsburgh, PA 15282-1530, USA*

This paper describes the logical structure of a hybrid expert-database system for microwave digestion: MWDXpert. It is a standard reference material (SRM) oriented expert system. Three hundred and twenty-two SRMs ranging from biological to geological materials, and 884 digestion procedures with reference to the SRMs are stored in a relational database. Procedures are summarized from hundreds of papers published from 1976 to 1998. The expert system assists the analysts in selection of the best match between the matrix of the sample and the representative SRMs. Digestion procedures applicable to the matrix are selected from the database and evaluated according to specific requirements. Procedures are further statistically analysed and ranked with principal component analysis. The procedure with the best performance is recommended to the analyst. Seven representative matrices based on SRMs have been tested with this expert system: NIST 1577 bovine liver, NIST 1572 citrus leaves, NIST 1566 oyster tissue, NIST 1633a coal fly ash, IFM 7 slag, NRC MESS-1, BCR 146 industrial sewage sludge sediment. The procedure recommended by the expert system can provide operating guidelines and a starting point for digestion optimization. After the first selected protocol with the recommended procedure, the expert system diagnoses problems that may be encountered during digestion. Possible causes and solution to the problems are provided. The digestion procedure is then revised according to the test results if necessary. The procedure combines data from the analyst and the database of methods and conditions to assist and refine sample preparation protocol and create robust optimal laboratory methods.

### Multicolumn automated HPLC method for the determination of molecular properties influencing the membrane transport of potential drug molecules

*Klara Valko, C. M. Du[1], M. H. Abraham[1], C. Bevan and D. Reynolds, Physical Sciences, GlaxoWellcome Medicines Research Centre, Stevenage, UK; [1]Department of Chemistry, University College London, London, UK*

A high-throughput method has been introduced for the determination of the chromatographic hydrophobicity index (CHI) using fast gradient-reversed phase HPLC. The CHI values were determined using the same method on various reversed-phase, cyclodextrin, nitrile, amino, diol and immobilized artificial membrane-coated columns. The various lipophilicity measures can be compared using the solvation equation that relates transport, distribution processes to various polar and non-polar molecular descriptors:

$$\mathrm{Log}\, SP = c + rR_2 + s\pi_2^{\mathrm{H}} + a\Sigma\alpha_2^{\mathrm{H}} + b\Sigma\beta_2^{\mathrm{H}} + vV_x$$

where $SP$ is a solute property (log\,$SP$ can be a CHI value) and the explanatory variables are $R_2$ (excess molar refraction), $\pi_2^{\mathrm{H}}$ (bipolarity/polarizability), $a\Sigma\alpha_2^{\mathrm{H}}$ and $b\Sigma\beta_2^{\mathrm{H}}$ (overall hydrogen bond acidity and basicity, respectively), $V_x$ (McGowan characteristic volume), and $c$, $r$, $s$, $a$, $b$ and $v$ are the constants that are characteristic to the particular mobile and stationary phase combination. The CHI values obtained on various HPLC stationary phases showed different rank of the model compounds; however, always significant correlation was found with the molecular descriptors. Setting up the solvation equation for five–eight very different stationary phase/mobile phase systems, we can calculate the molecular descriptors for new compounds from the easily measurable CHI values. With the help of the fast determination of the molecular descriptors, we can predict the transport/distribution behaviour of compounds in any systems for which the solvation equation is known (e.g. blood/brain barrier distribution, skin penetration, octanol/water partition, etc.).

### Mass encoding and qualitative analysis of combinatorial libraries

*David S. Wagner, Frank J. Schoenen, Robert Wilgus, Craig D. Wagner, Kenneth Kinsey and H. Mario Geysen, Diversity Sciences, GlaxoWellcome, RTP, NC 27709, USA*

Synthetic combinatorial libraries are fast becoming an important tool for lead discovery in the pharmaceutical industry. Advances in high-throughput screening coupled with combinatorial chemistry can significantly reduce the time to find lead compounds. The objective is to explore large parts of chemical space for a desired biological activity and to obtain a comprehensive SAR for the class of compounds synthesized. Encoding and decoding strategies are essential to combinatorial methods for the synthesis and analysis of combinatorial libraries. We developed a rapid and sensitive encoding methodology that utilizes stable isotopes and mass spectrometry. The ability of mass spectrometry to precisely determine the intensity of isotopic abundance provides a novel encoding strategy employing synthetically generated ratios of stable isotopes in a compound as the code. The encoding strategy can be used for generating small to very large libraries. The code can be cleaved after assay and analysed by mass spectrometry in an automated fashion. Mass encoding uses stable isotopes to produce distinct isotopic patterns in order to record the chemical synthesis. The code is either contained in the synthetic compound itself or on a separate molecular tag and analysed by mass spectrometry. We have successfully employed these encoding methods on solution phase, direct binding and lawn assays.

Because combinatorial chemistry generates huge numbers of chemical compounds, the number of samples requiring analytical characterization is tremendously increased. A significant problem with using biological data obtained on compounds made with minimal analytical data is in the interpretation of the results. Without analytical proof of the presence of a designed compound, it is dangerous to assume that the compound is inactive, as it may not have been present in the assay. Often it is difficult and time consuming to determine whether or not mass spectral peaks are due to the products, side reactions, reagents, solvents, or chemical impurities for a chemical reaction. We have developed several mass spectrometric methods to help ascertain all compounds that are cleaved from the resin bead or crown. The methods are invisible to the chemistry of the reaction and do not influence the chemical results. The procedures have been combined with our in-house computer algorithm (CAPTURE) and are fully automated. The method will be very useful for research in the areas of reaction screening, reaction database mining, monomer rehearsal and library synthesis.

### Implementation of PBMS in EPA's water programs

*Khouane Ditthavong, United States Environmental Protection Agency, Office of Water (4303), 401 M Street, SW, Washington, DC 20460, USA*

The Office of Water (OW) recognizes that implementing a performance-based approach to environmental measurement would require changes at the state and regional levels. OW, therefore, plans to offer a variety of communication and training tools to facilitate implementation. OW is working with the states through EPA regional offices to identify more accurately the type, frequency and amount of training that laboratory audit, program and enforcement personnel will require. Because the average turnover rate of National Pollutant Discharge Elimination System (NPDES) permit writers and drinking water laboratory certification officers is ~18 months, OW anticipates that it would be necessary to develop and maintain a continuously operating training program to adequately meet their training needs. OW will coordinate with EPA Regions, EPA's Environmental Monitoring Management Council, and the National Environmental Laboratory Accreditation Conference to develop and deliver communication and training programs to both the regulatory and regulated communities.

### How the National Environmental Laboratory Accreditation Conference (NELAC) standards meet the EPA's Office of Water's needs for a performance-based measurement system

*Jeanne Mourrain, US Environmental Protection Agency, Research Triangle Park, NC 27711, USA*

The purpose of the National Environmental Laboratory Accreditation Conference (NELAC) is to foster the generation of environmental laboratory data of known quality through the development of national performance standards for environmental laboratories to be implemented by state and federal accrediting authorities in a consistent fashion nationwide. The NELAC standards apply only where there are no existing regulatory requirements, i.e. federal regulatory requirements take precedence over the NELAC standards.

EPA's performance-based measurement system allows flexibility in method selection. To support this flexibility, the NELAC standards utilize an analyte-specific approach to proficiency testing. Under this approach, laboratory performance is audited against performance criteria for an analyte, regardless of the method used. Because OW's PBMS contains performance criteria, the NELAC standards reinforce OW's PBMS.

OW has recently privatized the water supply (WS) and water pollution (WP) performance-evaluation (PE) studies. This process valuates the performance of public and private sector laboratories to support the water programs. The NELAC standards are designed to reflect and complement the current requirements of the WS and WP studies.

### *In situ* monitoring of organic vapour by fibre optic FTIR

*Vane G. Smith, William R. Nicol Jr, Joseph A. Petitto, William F. Bryant Jr and Ronald J. Chernik[1], Indian Head Division Naval Surface Warfare Center, Indian Head, MD 20640, USA, [1]P. E., Envirovisions Corp., McLean, VA 22102, USA*

In an effort to passively monitor the organic solvent vapour concentrations in a large mixer vessel, a novel method utilizing Fourier transform infrared spectroscopy (FTIR) and mid-range infrared fibre optic cables was developed. This method was used in a 150-gallon horizontal mixer to determine the concentrations of ethanol and tetrahydrofuran (THF) vapour at various temperatures and locations in the mixer. A system will be described consisting of a FTIR spectrometer in an air-tight enclosure, connected via infrared-transmitting fibre optic cables to an infrared vapour probe inside the mixer. The probe was specifically designed and constructed to obtain the infrared vapour spectrum of any organic vapour/gases across its 10-cm path length. The system was remotely controlled by a computer.

Data obtained in three ethanol runs and two THF runs at various temperatures will be presented in this paper. The data were used to determine where and when during a mixing operation the organic vapour/air mixture was within explosive limits. Included will be techniques used to prepare vapour standards and calibration curves for quantitating the vapours in the actual test runs. Corroborating data generated by GC/MS analysis of the vapour samples drawn from the mixer during the runs will also be presented.

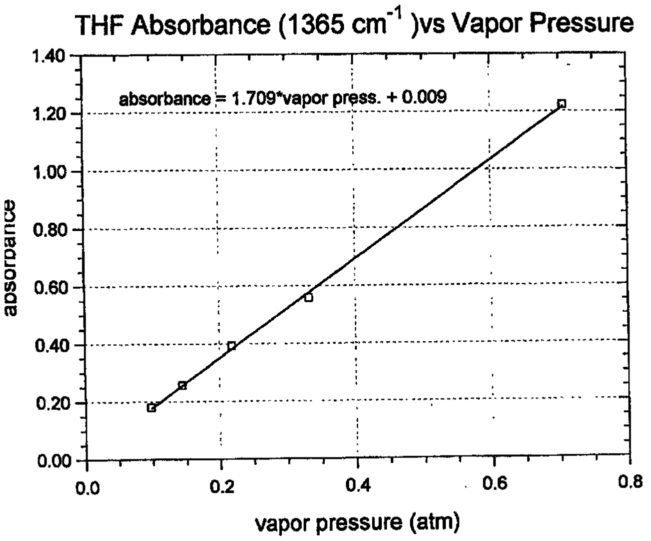

### On-line plant-extraction control by FTIR/ATR spectroscopy

*B. Arlitt, M. Kleimann and Heinz W. Siesler, Department of Physical Chemistry, University of Essen, D 45117 Essen, Germany*

For the isolation/production of pharmaceutical active ingredients, the technique of plant extraction plays an important role. This extraction can be performed discontinuously by maceration or digestion or continuously by percolation or in a Soxhlet. In the case of a continuous percolation, a high degree of enrichment can be achieved with water, alcohols or alcohol/water mixtures. Independently of the applied technique, a fast detection of the end-point is essential for the optimization of the extraction parameters. To this end, the content of active ingredient in the extraction solution has to be determined by a specific on-line method.

In the present paper, the potential of FTIR/ATR spectroscopy for on-line control of the extraction progress of Sennes leaves will be demonstrated. For this purpose we have modified an ATR-attachment as a flow-through cell and linked it up with a laboratory percolation system. The obtained results clearly indicate that the content of active ingredient in the aqueous solution can be readily determined on-line in the relevant concentration range of ~10–0.1 g/100 ml $H_2O$. The evaluation of the spectra has been performed by a univariate calibration as well as by a multi-variate chemometric model. The data obtained by mid-infrared ATR spectroscopy will also be commented on in terms of the comparative performance of near-infrared (NIR) light-fibre transmission and ATR spectroscopy based on a new sensor system.

### Development of an on-line, real-time monitoring system for the characterization of particulate matter

*Paul Nam, Peter Dawson, Shubhen Kapila, Philip Whitefield, Don Hagen and Steve Chelli, Department of Chemistry, Center for Environmental Science & Technology, Department of Physics,*

*and Cloud and Aerosols Science Lab, University of Missouri–Rolla, Rolla, MO 65401, USA*

A state-of-the-art particle characterization and monitoring system (mobile aerosol sampling system–MASS) developed at the University of Missouri–Rolla was interfaced to a mass spectrometer with an inductively coupled plasma ionization source. This tandem arrangement permitted real-time, on-line characterization of particulate matter in terms of particle size distribution and abundance as well as the elemental composition.

The performance of the integrated system was evaluated with two types of particle generator. The first source was a cross-flow nebulizer, which generated aqueous aerosols incorporated with target elements, especially metallic constituents. Real-time analysis of these aerosols with the integrated system confirmed the direct correlation between the narrow particle size distribution and elemental concentration profile. The second source was a hydrocarbon flame, which produced polydispersed combustion particles. On-line determination of metal concentration in the size-segmented particles was found to relate well with the combustion stoichiometry.

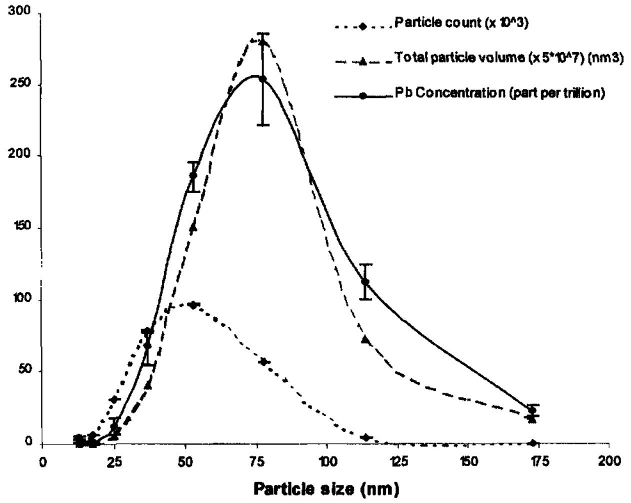

*Real-time, on-line characterization of nebulized aerosols by the particulate monitoring system.*

### Sequential injection method for monitoring water in the energy co-generation system from a MW incinerator

*F. M. Bauzá de Mirabó, R. Forteza, V. Cerdà and M. T. Oms*[1]*, Department of Chemistry, University of the Balearic Islands, Carretera de Valldemossa, km 7.5, E-07071 Palma de Mallorca, Spain;* [1] *TIRME SA, Carretera de Sóller, km 8.2, E-07120 Palma de Mallorca, Spain*

In the co-generation system of the MW incinerator, it is of great importance to control several parameters, e.g. conductivity, acid conductivity, $SiO_2$, $PO_4^{-3}$, Fe, pH, hydrazine and $NH_4^+$. As carrying out the analysis is tedious, flow methods are helpful for this purpose. Therefore, a sequential injection method has been developed because of its compactness and low reagent consumption.

In this first work, the parameters considered are hydrazine and ammonium. Hydracine is determined using *p*-dimethylaminobenzaldehyde in the range 0.05–2 mg/l $N_2H_4$, where 0.017 mg/l is the detection limit and the relative standard deviation is 1.53% (1 ppm $N_2H_4$ and $n = 10$). Ammonia is determined using the salycilate method in the range 1–18 mg/l $NH_4^+$, the detection limit being 0.08 mg/l and the relative standard deviation is 3.5% (10 ppm $NH_4^+$ and $n = 10$).

This method has been applied to real samples from the Son Reus incineration plant (Palma de Mallorca, Spain). The results obtained have been compared with those ones obtained in batch methods with satisfactory results.

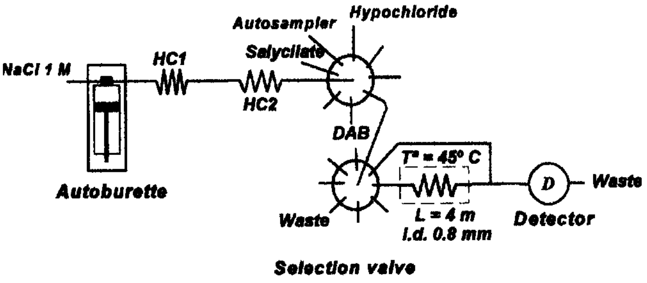

*System configuration for the determination of hydrazine and ammonium.*

### Use of laser-based polarimetric detection in process control and quality assurance applications

*Donald R. Bobbitt, Brandon Mosley, Gary W. Yanik*[1] *and Mark S. Alper*[1]*, Department of Chemistry and Biochemistry, University of Arkansas, Fayetteville, AR 72701, USA;* [1]*PDR-Chiral, 3 Old Meadow Way, Palm Beach Gardens, FL 33418-3720, USA*

Many important problems in the biotechnology and pharmaceutical industries require explicit information about the chirality of one or more materials involved in a process or formulation. This information could be used to directly assess enantiomeric purity, or, it could be used to identify changes occurring at, or near a chiral centre. Direct observation of the chirality of a given compound under the sample constraints of HPLC or other modern separation modalities has been problematic, in spite of the advantages that could be realized from such an approach. Recently, a new, turn-key, laser-based polarimetric detection system has been introduced which provides state-of-the-art rotational sensitivity while combining operational robustness with simple operational characteristics. In this presentation, this unique detection approach will be applied to problems related to either process control or quality assurance applications.

In the synthesis of chiral materials on a large scale, intimate knowledge of the optical activity of the reaction mixture allows one control over the reaction process, in order to optimize the process in terms of either yield or cost considerations. As an example, an enzyme-catalysed conversion process will be followed using laser-based polarimetric detection. In this application, the substrate and product possess different specific rotations allowing one to assess the progress of the reaction by monitoring the net optical activity of the sample introduced into the polarimeter via flow injection analysis.

Alternately, in the analysis of a formulated product involving a chiral drug substance, measurement of the chirality of the sample after separation provides a direct and simple way to assess degradation and/or formulation consistency. Several examples will be presented involving the analysis of antibiotic formulations used for the treatment of infection in humans and/or animals. In each example, determination of specific rotation for eluting compounds will be shown to provide a specific and sensitive qualitative indicator which can be used for identification of the antibiotic analogue.

### LIMS: the need for flexibility in a regulated forensic environment

*Trevor de Silva, HFL, Newmarket Road, Fordham, Cambridgeshire CB7 5WW, UK*

In 1990 we installed our first LIMS. With its extensive use of bar codes, sample tracking and instrument interfacing, the system was considered a great success. Staff were able to review chromatographic data from any PC in the laboratory and enter their interpreted result. Other results were uploaded to the servers where background processes would transfer them to the database. Additional reports allowed all chromatographic and interpretative results for a single sample to be reviewed from a single screen.

The system was a heavily customized version of an 'off the shelf' package and ultimately, this was its greatest weakness.

HFL is a dynamic laboratory, business rules and procedures change regularly. The LIMS needs to be able to adapt to these changes, while still adhering to the constraints of the forensic environment.

- Chain of custody.
- Multi-level result review.
- External regulatory compliance.

HFL

In 1995, we decided to replace our LIMS, and we had the opportunity of being involved in the development of a new system. This new system needed to provide all the functionality of the existing system while allowing for continued configuration changes to our business rules without the need for time-consuming customization.

This paper will discuss the following.

- Justifying the replacement.
- Working with a vendor to develop a new system.
- Migration.
- How we feel that our demanding requirements have been satisfied.

### Implementation of rapid result management systems in the metals industry

*Emile Muller, Marc A. Bassin, Jean-Pierre Troyon, Paul Nowak and Barry W. Adamson, ARL Applied Research Laboratories S.A., En Vallaire Ouest C, CH-I 024 Ecublens, Switzerland*

A wide variety of result management systems exist for use with analytical instruments, the majority of which come under the generic term 'LIMS' (laboratory information management system). These systems are often both powerful and extremely comprehensive which, paradoxically, can create difficulties when attempting to apply them in certain specific application areas.

One such application area is process control in the metals industry.

The need to merge analytical results from an optical emission spectrometer, an XRF system and combustion analysers, and transmit the merged data back to the process is a typical requirement in these environments, This is not a particularly demanding requirement in itself, and many conventional LIMS systems would be considered as an 'overkill' solution. However, the crucial difference between this application and others is the fact that there may be hundreds of tonnes of molten metal awaiting the merged analytical result. When considering the energy costs related to maintaining metal in a molten state, then speed of operation is clearly the critical performance criteria for the merging and routing software in this application area.

The system presented here, ARMS (ARL result management software), shows how it is possible to design a system for result merging and routing which takes account of the speed requirement as the number one priority. Examples of system implementation in a variety of metal industries will be given, and the accrued benefits from the faster operation will be reviewed.

### Quarter of a century of the LIMS evolution

*Thomas W. Dollarhide and Jay Chrystal, PURVIS Systems, 1272 West Main Road, Middletown, RI 02842, USA; [1]Chemserve Environmental Laboratory, 317 Elm Street, Milford, NH 03055, USA*

Advances in commercial software development tools have resulted in the availability of numerous LIMS products. The majority of these products are not well suited to the needs of smaller laboratories, both in terms of functionality and affordability. PURVIS Systems recognized this void. Leveraging new technology against our 25 years of experience in developing custom LIMS solutions for varied industries, PURVIS developed an affordable LIMS product to meet the needs of one specific market—the small to mid-size environmental lab. The first half of this paper details PURVIS' LIMS experience and the development of the 'off-the-shelf' product. This experience is coupled with the perspective of the end user, the environmental lab, to provide a total picture of LIMS in an environmental lab setting. In June 1997, Chemserve was the first Beta site that installed the PURVIS LIMS. Chemserve's experience is provided in the second half of the paper.

Interviews with environmental labs in the New England area were conducted to develop a software specification. Although environmental labs perform similar analyses, they operate very differently from one another. This is the single greatest challenge faced in the development of a LIMS product. Using past industry experiences combined with end user inputs, a software package was developed that meets the majority of both the analytical and operational requirements of the environmental laboratories. In order to accommodate as many of the requirements mentioned by the diverse laboratories interviewed as possible, much more functionality was included than was originally envisioned. This increased development time and costs considerably from the original plan. Requirements included compatibility with existing analytical and business operations, improved laboratory efficiency, better quality control and affordability.

Development of the PURVIS LIMS software product using standard programming languages and tools enables the product to be customized by PURVIS or qualified laboratory personnel if further functionality is required.

This portion of the paper provides a practical perspective from a laboratory that was the first to use PURVIS' system and one that worked closely with PURVIS during Beta development. Our perspective includes mistakes that were made along the way, some of the dos and don'ts of installation/implementation, and some of the triumphs achieved after implementation.

Some of the key areas covered include: (i) how our laboratory uses the system; (ii) what to plan for prior to implementation; (iii) avoiding the possible pitfalls; (iv) what and how much training to plan for; and (v) the extent to which a LIMS product can improve lab efficiency.

This paper will be an information source for any small to mid-size environmental lab that either does not currently have a LIMS or has an older, obsolete system. We will attempt to provide a true laboratory perspective addressing key issues.

### Training is the key to a successful LIMS implementation

*Cynthia Pease-Nienart and Peter G. Berthrong[1], C.A.N. Associates, 603 Boozer Lane, Neshanic Station, NJ 08853, USA; [1]ISO Validation Services, 20 Old Republic Lane, Marlton, NJ 08053, USA*

The key to a successful implementation is having a staff that is properly trained and confident in the use of the laboratory information management system (LIMS). The training process involves four important steps: detailed outline; workbook; lectures; and workshops.

The first step of an effective training course is to interview the customer on the sample flow in the lab and create an outline that correlates the sample flow with the LIMS. A flow chart illustrates the steps in an efficient condensed format.

In the second step, once the detailed outline is created, a workbook with screen captures from the LIMS system is generated. The workbook format depends on the customer's requirements. Several different formats will be presented, and the pro and cons of each type discussed. In general, it has the screen captures with a short discussion on the general use and operation of the screen and a space for student notes. The discussion can be presented in the form of description and action columns or a detailed field description. The workbook can reference company SOPs and other pertinent information, e.g. naming conventions, etc.

Detailed lectures are presented from this workbook in the third step. These lectures cover the 'sample life cycle' and include login, receipt, testing, review of the test data, approval and reporting. At the end of the course, the student should have entered data in corresponding screens and be familiar with the flow of the sample in the LIMS system.

Reinforcing the lecture material with workshops is the final step. The workshops are hands-on examples that repeat the important topics in the lecture material. Students perform the workshops during class time so the instructor is available to help them. They also retain the workshop questions and answers as a future reference.

The training process improves communication between the supervisors and the lab people. It also educates employees, reduces the LIMS implementation cycle time and is the key to a successful LIMS.

### Computer science-based solution for a water quality control laboratory management

*Mohammed Elmghari Tabib, Abdelmjid Amghar, Mahjouba Bourziza, Brahim Kers and Lahoussaine Echihabi, ONEP Quality Control Laboratory, BP Rabat Chellah, Rabat, Morocco*

The National Office of Potable Water (ONEP) is a public body in charge of the production, control and distribution of potable water throughout the Kingdom of Morocco.

The ONEP Central Laboratory and a network of 46 decentralized laboratories are in charge of quality monitoring of produced and distributed water, and also pollution control of resources intended for drinking water production. In order to perform this activity, ONEP Laboratories follow a variety of sampling points and type of samples (treated water, raw water, waste water, sludge from sewage works, treatment reagents etc.) depending on the objective of control. The variety of biological and physicochemical analysis as well as the significant amount of data generated by ONEP laboratories have speedily imposed the need for a data computer management.

The ONEP Central Laboratory has developed a computer science-based application for laboratory management (GDAL). This application was devised and adopted to satisfy the present and future needs of the Central Laboratory. The pro-software was developed under Power Builder within windows environment (NT

and 95). It manages more than 3500 sampling sites throughout Morocco, that is more than 30 000 samples 1 million of analytical data, and enables to edit more than 20 types of analysis reports. This system is intended for 21 users simultaneously and allows the management of analytical data of the central and decentralized laboratories.

The system is developed into modules covering all sample life cycle as below:

- analysis campaign planning;
- management of samples taking missions;
- samples reception;
- analysis;
- invoicing;
- consultation and printing;
- processing analytical data provided by the system.

GDAL also comprises the laboratory administrative and stock management, and accounting.

The purposes of this application are as follows.

- To enable each user to have access to the information about samples analysis in real time.
- To automate everyday tasks of the laboratory.
- To ensure the follow up of all the information concerning the analysis from planning the sampling to edition analysis report.
- To impove the accuracy of the results by automatic quality control procedures.

### The determination of total nitrogen and trace metals in soil using continuous flow microwave digestion

*Christopher J. Mason, Mark Edwards and Philip G. Riby, School of Chemical and Life Sciences, University of Greenwich, Wellington Street, Woolwich, London SE18 6PF, UK*

A fully automated method has been developed for the determination of the total nitrogen content of soil using microwave-assisted Kjeldahl digestion. The system exhibits wide bore continuous flow technology, producing a sample ready for analysis in less than 15 min. Using a double pumping pressurization system, back pressures of up to 300 psi can be achieved, whilst maintaining steady even flow. The pressurization system exhibits blockage-free operation for slurries up to 2% for 1000 μm particle sizes. A constant flow rate allowing digestion times in the microwave to be reproducible is achieved using this pressurization system for a wide range of soil types that produce different pressure profiles. Analysis of digested samples for nitrogen content takes place colorimetrically using a flow injection system which makes use of the Berthelot reaction. The detection limit of the system for nitrogen is 6 ppb. Rapid analysis can be carried out at a rate of $55\,h^{-1}$ using microwave energy to accelerate colour formation, compared with $30\,h^{-1}$ using conventional thermal energy. The above system can also be adapted to digest soil in aqua regia for trace metals analysis. The digestion procedure in this instance takes less than 10 min, achieving a 95–105% extraction of aqua regia-soluble metals.

### Extensions of Method 7473 for mercury analysis

*Helen M. Boylan, H. M. 'Skip' Kingston, Robert C. Richter and Scott Rice[1], Duquesne University, Department of Chemistry and Biochemistry, 600 Forbes Ave., Pittsburgh, PA 15282-1530, USA; [1]SE Technologies, 98 Vanadium Rd., Bridgeville, PA 15017, USA*

EPA Method 7473 is a new technique, based on traditional methodologies, for the analysis of total mercury in environmental matrices. The method functions on the basis of thermal decomposition, amalgamation and atomic absorption spectrometry. Recently developed instrumentation integrating these concepts has eliminated the reproducibility and interference issues associated with original thermal decomposition techniques. With Method 7473, sample preparation and analysis are essentially integrated into a single analytical instrument, allowing for direct analysis of solid samples. Direct analysis gives Method 7473 the capability to be applied in either laboratory or field settings. Field results from this technique have been shown to surpass traditional laboratory results in terms of both precision and detection limits. Validation data for the method using a variety of certified samples, as well as actual field results, have been previously presented.

While Method 7473 has been validated for traditional environmental samples, there is a need to extend this technique for the analysis of unique matrices and mercury species. Proposed emission regulations have sparked interest in analysing mercury content in coal, crude oil and gasolines. Other samples of interest include air filters, paint chips and fish tissue. Procedures based on Method 7473 have already been successfully applied for the analysis of mercury in coal, Teflon-coated quartz filters and a variety of seafood samples. Method development for these and other unique matrices will be discussed.

The EPA has recognized the need for a reliable measurement technique for mercury speciation. Use of thermal programming for the analysis of operationally defined mercury species has been reported. Unusual peak shapes resulting from analysis of certain samples by Method 7473 may suggest the presence of mercury species with different thermal characteristics. Investigation of the thermal properties of target mercury species, including organomercury species, will be described in this paper. By extending Method 7473 to include thermal programming, the authors envision development of a technique capable of on-site screening of operationally defined mercury species.

### Development and application of a chromium speciation method using an automated liquid handling system with ICP-MS detection

*Mary Kate Donais and Tom Rettberg, VG Elemental, 27 Forge Parkway, Franklin, MA 02038, USA*

The speciation of inorganic chromium in environmental samples is necessary to accurately assess pollution levels. Of the two chromium oxidation states, Cr(VI) is a known carcinogen, while Cr(III) is an essential element. Due to the widely different toxicity of the two forms of chromium, total chromium measurements can not be used to determine actual environmental impact. The speciation of chromium in environmental samples is therefore necessary to accurately assess pollution levels.

An automated liquid handling system, the PrepLab, together with inductively coupled plasma-mass spectrometric detection (ICP-MS) were investigated for quantification of Cr(III) and Cr(VI) in liquid samples. The separation of Cr(III) and Cr(VI) using various chromatographic resins and mobile phases was investigated. An application has been developed using a solid phase chelation resin with an iminodiacetic acid functional group.

The resin is hand-packed in a low-pressure glass column ($3 \times 25$ mm). Samples are introduced to the column by means of two dual-channel peristaltic pumps and a dual six-port injection valve. All control of the liquid handling system is within the ICP-MS software.

Data will be presented for the analyses of real water samples. Studies of possible interfering species and various method figures of merit will be discussed in this paper.

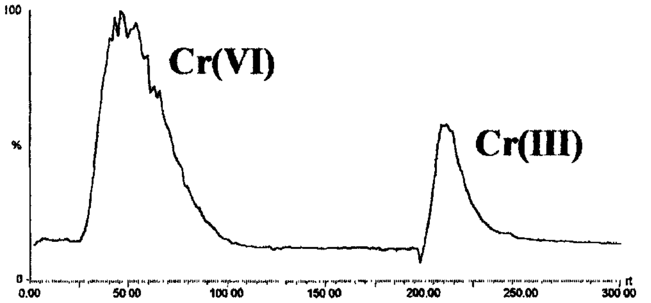

*Separation of Cr(VI) and Cr(III) using solid phase chelation column and ICP-MS detection.*

**Determination of chromium species by flow injection analysis with dual-membrane extraction and ICP-MS detection**

*Ray Thibault, Mary Kate Donais*[1] *and Stuart J. Chalk, Department of Natural Sciences, University of North Florida, Jacksonville, FL 32224, USA;* [1]*VG Elemental, 27 Forge Parkway, Franklin, MA 02038, USA*

The accurate speciated analysis of chromium has become one of the most important, yet elusive, analytical problems to environmental chemists. Speciated analysis of chromium in the +3 cationic oxidation state, and in the +6 anionic oxidation state [as chromate ($CrO_4^{-2}$)] is conceptually simple. The nature of the two species provides the obvious separation mechanism of ion exchange. However, the kinetics of the Cr(III)/Cr(IV) redox couple in aqueous solution are fast, and thus the removal of one oxidation state from solution causes reduction or oxidation of the other species to occur. The result is inaccurate speciated information due to species interconversion during analysis.

To combat this, we propose a novel membrane reactor that removes the two species from aqueous solution at the same time. Chromium(III) is removed by cation exchange through a Nafion membrane. At the same time, chromium, as chromate, is removed from solution by anion exchange through a poly-4-vinylpyridine membrane, or commercial anion exchange membranes. If extraction conditions are optimized correctly, removal of each of the species can occur at the same stoichiometric rate, the equilibrium is not disturbed, and an accurate estimate of the relative amounts of each species can be made.

This dual-membrane reactor is implemented in a flow injection analysis manifold as shown in the figure below. With this device, not only can separation of the species occur but preconcentration of the two forms can be realized by stopping the flow of one, or both, acceptor streams. Initial experiments were performed using flame AAS detection, and then the apparatus was adapted for low-level ICP-MS detection.

In this paper, we will discuss the design and operation of the reactor, detail some of its characteristics in terms of transport efficiency, discuss integration of the reactor into an automated liquid handling system, and show the analysis of chromium-contaminated water samples using this approach.

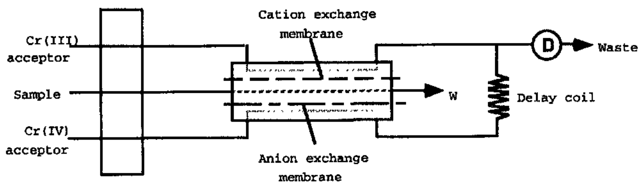

**Automation or manual titration? The comparison of a manual titration with a visual end point and an automated titration with a potentiometric end point in a cerium redox titration**

*Johanna M. Smeller, National Institute of Standards and Technology, 100 Bureau Drive, Stop 8393, Gaithersburg, MD 20899-8393, USA*

A series of repetitive titrations can be time consuming. Many visual indicators used in manual titrations are unstable and require operator experience. Automation, on the other hand, saves time.

The concentrations of two cerium solutions were determined using a redox titration procedure. The titrant, ferrous ammonium sulphate (FAS), was standardized daily using three metal calibrant solutions. The sample preparation involved adding sulphuric acid and silver nitrate, heating to a vigorous boil, and adding ammonium persulphate. Then the solutions were titrated using either a visual end point or a potentiometric end point.

With the visual endpoint determination, the indicator ($FeSO_4$ $7H_2O$ and 1,10 phenanthroline in $H_2O$) was added and the FAS was added gravimetrically to the end point, indicated by a colour change from light green to light orange.

With the potentiometric end point determination, ~96% of the FAS was added gravimetrically and the remainder of the FAS was titrated volumetrically to a potentiometric end point using an autotitrator. A combination platinum electrode was used with an automated titration system. Using a linear titration method, 0.01-ml increments of dilute FAS were added. The end point was determined as the maximum first derivative.

The cerium results obtained from the manual and automated methods agree, but the potentiometric end point method yielded results six times more precise and saved time.

### Extraction of elemental and molecular information from capillary electrophoresis plasma mass spectrometry

*Vahid Majidi, Robert Steiner, Wei Hang and David Wayne, CST-9, MS k484, Los Alamos National Laboratory, Los Alamos, NM 87545, USA*

During the past few years, a number of researchers have successfully combined capillary electrophoresis with inductively coupled plasma sources and mass spectrometric detection to collect information with regard to elemental speciation in a given sample. Although partial molecular information can be acquired from the relative migration time, no direct evidence can be obtained with these systems. This is because of the energetic nature of the ICP, where all compounds are reduced into their constituent elements and ultimately ionized.

Our work focuses on collecting molecular and elemental information from a given sample. One approach is to use two independent techniques for elemental and molecular information. To meet this challenge we have used CE-ICP-MS to gather elemental information on various samples. These data are then augmented with full molecular information from either electrospray mass spectrometry or partial molecular information from UV-VIS absorption spectrometry. Although extremely powerful, this approach is limited because of low throughput and solvent incompatibilities.

Another approach for concurrent collection of molecular and elemental information is to use modulated plasmas. In this paper we will outline our current work on low/high-energy plasma systems that have the analytical potential for simultaneous molecular and elemental speciation with capillary electrophoresis separation. Depending on the temporal behaviour of modulated plasmas, it is possible to generate elemental information, molecular ion information and fragmentation patterns. The advantages and shortcomings of the above approaches will be discussed in this paper.

### Determination of organochlorines in biological samples using focused microwave-assisted extraction and gas chromatography with electron capture detection

*S. Thompson, H. Budzinski and P. Garrigues, LPTC, UPRESA 5472, Université de Bordeaux 1, 351 Cours de la Liberation, 33405 Talence, France*

Organochlorine compounds, e.g. polychlorinated biphenyls (PCBS) and chlorinated pesticides, e.g. DDT and lindane, are ubiquitous pollutants in the environment which are subject to strict regulation. The US Food and Drug Administration (FDA) among others, has set tolerance levels for PCBS in various foodstuffs, e.g. 2 ppm in shellfish and fish. This paper presents a new methodology for the determination of organochlorines in environmental biological samples.

The procedure involves the use of focused microwave-assisted extraction. This novel technique is rapid (10 min) and uses a reduced volume of solvent compared to classical extraction methods, e.g. Soxhlet. The extraction is performed on the freeze-dried sample in an organic solvent. The extract is purified by concentrated sulphuric acid treatment and chromatography on silica gel. The PCBs are separated from the chlorinated pesticides by HPLC on an amino-silane column in normal phase mode. The extracts are analysed by gas chromatography with an electron capture detector. Several internal standards are used for quantitation.

This method was validated with a standard reference material from NIST, SRM 1588, Organics in Cod Liver Oil. The compounds have good recoveries ranging from 80 to 120%. The reproducibilities are also good with an average standard deviation of <10%.

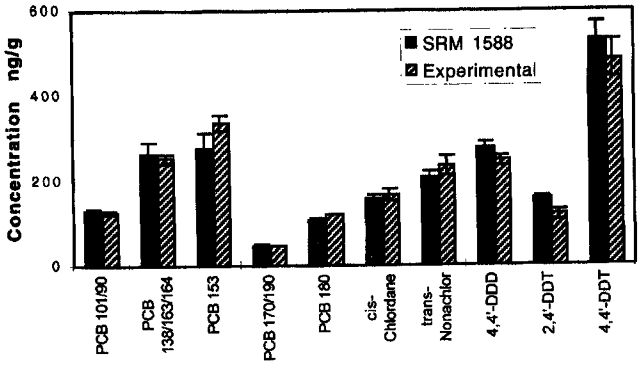

*Validation results for SRM 1588.*

### Multicomponent SIA determination of phenols in waters by on-line extraction and preconcentration

*Victor Cerdà, Andreu Cladera, Manuel Miró and J. Manuel Estela, Department of Chemistry, University of Balearic Islands, 07071 Palma de Mallorca, Spain*

An automatic sequential injection analysis (SIA) set-up for the determination of phenolic compounds (2-, 3- and 4-nitrophenol) in water was designed and evaluated. The system performs an on-line phenol extraction from the aqueous media to an organic solvent, which enables the analyte isolation and preconcentration. The extracted phenols are on-line back-extracted into a small volume of NaOH solution and conducted to a spectrophotometric detector for quantitative analysis. This methodology is based on the formation of a thin organic solvent layer adhered to the PTFE surface of the extraction coil. In the present work, no flow-reversal was performed in the extraction and back-extraction steps.

Spectrophotometric detection (photodiode-array detector), multi-linear regression algorithms and first derivative spectra techniques were evaluated in order to perform multicomponent determinations of phenol mixtures. Concentrations within the range 0.5–4.5 mg of 2-nitrophenol $l^{-1}$, 0.7–11.2 mg of 3-nitrophenol $l^{-1}$ and 70 930 µg of 4-nitrophenol $l^{-1}$ have been determined at a frequency of 4 samples/h, analysing the three compounds simultaneously.

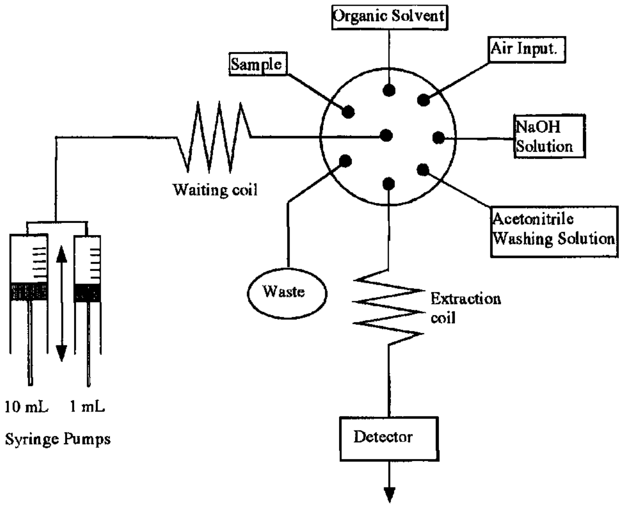

*SIA manifold used for the extraction and preconcentration of phenols.*

### New sample preparation procedure for the determination of polycyclic aromatic hydrocarbons and polychlorinated biphenyls in surface waters

*T. Górecki, L. Wolska*[1]*, K. Galer*[1] *and J. Namieśnik*[1]*, Department of Chemistry, University of Waterloo, Waterloo, ON, Canada N2L 3GI;* [1]*Chemical Faculty, Technical University of Gdańsk, Narutowicza 11/12, 80-952 Gdańsk, Poland*

Suspended matter present in surface waters in a wide range of concentrations makes reliable determination of polycyclic aromatic hydrocarbons (PAHs) and polychlorinated biphenyls (PCBs) a very difficult task. Analytical and technical problems caused by the suspended matter affect both accuracy and precision of analyte determination in the dissolved phase. Because of their low volatility and hydrophobic character, both PAHs and PCBs are easily adsorbed by suspended particulate matter (SPM), as well as absorbed by humic and fulvic acids, lipids and proteins, forming dissolved organic matter. Concentration differences between SPM and solution due to preferential adsorption can reach a factor of $10^5$ or more. The presence of SPM during sample transport and storage can reduce PAH recoveries by 20–70%.

Filtration under gravity, by applying vacuum or pressure, as well as centrifugation, are typically used to eliminate SPM from samples. Clogging, analyte adsorption on the filter and on the layer of particulate matter formed on the filter are the main problems encountered during filtration.

A new procedure for the analysis of PAHs and PCBs in water containing SPM was developed. A specially designed filtration vessel coupled directly to an SPE cartridge was used for this purpose. The vessel contained a 0.7 µm filter with ~4 g of high-density glass beads on top of it to prevent clogging. The filtrate was introduced directly to the SPE cartridge. SPM separation and analyte isolation/concentration were carried out in a single step. Both the SPE cartridge and the suspended matter collected on the filter were solvent extracted, and analyte recoveries were determined. Recoveries of PAHs with the highest aqueous solubilities from the filtrate ranged from 80 to 100%, while those for PAHs with the lowest solubilities did not exceed 20%. Total recoveries of PAHs from surface water containing 21 mg/l SPM ranged from 82 to 127%. PCB recoveries from the particulate matter reached more than 10%, while those from the filtrate ranged from 26 to 57%. Total PCB recoveries ranged from 39 to 70%.

### Semi-preparative sample purification with SFC

*Alan Hamstra, Gilson, 3000 West Beltline Highway, Middleton, WI 53562, USA*

Semi-preparative sample purification with supercritical fluid chromatography (SFC) has the advantages of speed, selectivity, minimal collection volume and low mobile phase cost. Collection on this scale can yield 500 mg of sample per hour with a recovery of 90%.

Injection volume is limited for the following reasons. Samples are loaded onto the chromatography column dissolved in solvent other than the mobile phase, this may significantly alter chromatographic resolution. The liquid carbon dioxide in the injection loop becomes gas instantly when the injection valve shifts from the inject position to the load position, limiting the volume of injection loop that can be used safely. Samples should be loaded as concentrated as possible for maximum loading capacity.

Samples are collected into vials with volumes from 20 to 250 ml, depending on the system flow rate. Carbon dioxide volume increases 500-fold and cools significantly when it changes state from liquid to gas. Without precautions, pure sample is blown out of the collection vial or carried away with the carbon dioxide gas. Positioning of the collection probe within the vial significantly influences sample recovery.

The solvating strength of supercritical carbon dioxide depends on its density. After the final restriction valve, the density of carbon dioxide drops to atmospheric pressure, and the sample is no longer in solution. Samples that are purified on the chromatography column may precipitate in the pressure regulator, on the inside of tubing or plug the restrictor. Addition of modifier solvent after sample detection keeps the sample in solution and increases sample recovery.

In this paper, suggestions and examples to help optimize sample loading and collection conditions for 0.46 cm and 1.0 cm I.D. columns with flow rates from 3.0 ml/min to 9.0 ml/min are presented.

### An on-line measurement system for accurate quantitative monitoring of powdered materials using near-infrared spectroscopy

*Arnold J. Eilert, Donald E. Muller and John Beauchaine, Bran + Luebbe, 1025 Busch Parkway, Buffalo Grove, IL 60089, USA*

Near-infrared (NIR) spectroscopy is an extremely useful technique for rapid quantitative analysis of a wide variety of sample types with little or no sample preparation. Consequently, NIR has been increasingly used on-line for real-time process monitoring/control. For certain products (i.e. clear liquids), the material can be spectroscopically interrogated in transmission mode while simply being allowed to flow through an appropriate sample chamber. However, many other product types require special sample handling. Powdered materials have, for many years, been successfully analysed with laboratory/at-line systems using near-infrared diffuse reflectance spectroscopy. This has been performed with accessories that properly contain the sample and present it in a controlled manner to the analyser. However, rugged on-line measurement systems have recently been developed that provide automated powder transport with controlled filling of an appropriate sample chamber. These systems are designed to provide analytical precision with powders that rival NIR analyses performed in the laboratory. The similarity of optics and sample presentation used in the on-line systems to laboratory/ at-line systems enables calibration transfer from lab to process. Successful examples of on-line NIR analysis of powdered food products/ingredients will be presented in this paper.

### High-speed hyphenated gas chromatography followed by chemometric analysis of environmental pollutants

*Robert E. Synovec, Carsten A. Bruckner, Bryan J. Prazen and Carlos G. Fraga, Center for Process Analytical Chemistry (CPAC), Department of Chemistry, Box 351700, University of Washington, Seattle, WA 98195, USA*

Gas chromatography (GC) is generally practised to achieve baseline resolution of the analyte(s) of interest. While the current practice has been successful, the resolution requirement severely limits the applicability of GC in the analysis of complex environmental and industrial samples. Furthermore, the necessity to fully resolve all analytes of interest is often impractical, and significantly lengthens chromatographic run times. In order to reduce analysis time and obtain useful chemical data, it has become imperative to investigate novel approaches of applying chromatography.

We are bridging the fields of high-speed multi-dimensional gas chromatography and chemometric analysis. We have developed high-speed comprehensive GC × GC and GC/time-of flight MS instruments, providing rapid separations of complex samples. When coupled with chemometric analysis, these two-dimensional analysers hold considerable promise as powerful tools to address needs in environmental and process analysis. We report advances in three areas of chemometric data analysis: first, retention time standardization, which objectively corrects for run-to-run retention time variation; second, simultaneous quantification of several analytes in GC × GC data in the presence of unknown interferences; and third, novel pattern recognition methods for rapid classification and screening applications. We also report upon the hyphenation of water liquid chromatography (WLC) to high-speed GC, referred to as comprehensive WLC × GC. WLC separations require only water as the mobile phase, thus excluding the need for organic solvents in the chemical analysis. This significantly reduces maintenance costs while enhancing safety, thus extending LC applicability to remote environmental and process monitoring.

### Automatic elemental analysis—a vital tool for coal, coke and graphite characterization

*Henry Hancock, Liliana Krotz[1], Luigi Ragaglia[1] and Franca Andreolini, CE Elantech, 170 Oberlin Ave., Suite 5, Lakewood, NJ 08724, USA; [1] ThermoQuest Italia-CE Instruments, Strada Rivoltana, 20090 Rodano (Mi), Italy*

The characterization of coal, coke and graphite elemental composition and the heat value are becoming very important, they are essential to the classification and evaluation of this type of material. By its nature, coal is a non-homogeneous and unstable substance. It changes in quality as soon as it is mined and continues to react with oxygen at varying rates depending on moisture, mineral water, etc. to which it is exposed. The EA 1110 elemental analyser used for this work has proven to be the most reliable instrument for this application and copes with all requirements of modern laboratories, e.g. short analysis time, accuracy, reproducibility and low cost per analysis.

For CHNS determination, the instrument operates according to the dynamic flash combustion method, GC separation and thermal conductivity detection, while for oxygen determination, the system operates in pyrolysis mode. The heat value, that is the only quantitative measurement of the energy output, can be determined by measurement of the CHNS and oxygen content, and using a modified Dulong–Petit equation. The fully automated procedure can provide for CHNS determinations of up to 196 unattended samples in less than 10 min each, and for oxygen determination in less than 5 min each.

A series of experimental results obtained from the analysis of different samples is shown and discussed in relation to the simultaneous CHNS analysis, the determination of oxygen, heat value calculation, and the outstanding day to day reproducibility and repeatability of data.

### Development of an electronic tongue

*Suzanne K. Schreyer and Susan R. Mikkelsen, Department of Chemistry and Biochemistry, University of Waterloo, Waterloo, Ontario, Canada N2L 3G1*

The development of electronic noses (gas sensor arrays) and electronic tongues (liquid sensor arrays) has been made possible by the advent of computer software for multivariate analysis. Gas sensor arrays have become widely used as mimics for the mammalian olfactory system in a variety of applications, e.g. in analysis of pig malodours and for cluster analysis of flavour samples. Similar concepts are involved in electronic tongues, but in these cases, the analysis media are liquid. For example, in the analysis of food samples, e.g. milk and fruit juices, voltammetric techniques have been used to characterize the samples, in conjunction with principal components data analysis. In particular, pattern recognition techniques are commonly used.

Based on the work of Winquist *et al.* [*Anal. Chim. Acta*, **357** (1997) 21–31], we have developed an electronic tongue for the classification of liquid samples, using factor analysis to reduce the matrix to significant factors, and then cluster analysis to classify the various sub-populations. Data for the matrix were collected by either square wave or normal pulse voltammetry using working electrodes of platinum, glassy carbon and gold. The liquid samples tested were differing types of complex media, including wines, beers, fruit juices, coffees and dairy products, without any pre-treatment of samples. Factor analysis using both the MATLAB and NIPALS (non-iterative partial least squares) algorithms were used to generate the scores, and finally, cluster plots were generated to show the sub-populations. These plots show that square wave voltammetry with a platinum working electrode is effective in discriminating the various liquid samples. Training and validation results will be presented in this paper.

### Implementation of and applications for a chromatography database

*R. W. Albrecht and L. Wilhelm, LC Resources, 2930 Camino Diablo #110, Walnut Creek, CA 94596, USA*

Many data-processing applications are most easily implemented using a relational database, and this is the case for chromatography data. The creation of, and specific applications for chromatography databases are covered in this paper. General topics of interest include: the best database layout, importing data files, report generation, queries, LIMS system connectivity, and manual and automated data entry. Software used for automating the creation and maintenance of chromatography databases will be presented.

### LIMSymphony—LIMS implementation methodology

*Ron Sella, LabVantage Solutions, 245 Highway 22 West, Bridgewater, NJ 08807, USA*

In order for a LIMS implementation to be successful, there must be a well-defined methodology for implementation. This paper addresses one highly successful approach used by LabVantage Solutions—LIMSymphony.

LIMSymphony has been refined over many implementations to take account of the needs of many different types of laboratory, and is delivered with installation of LabVantage Solutions LIMS.

The process contains 10 phases beginning pre-sale with a certification of the scope of the project, and ending with a transition to LVS support services.

A summary of the steps is as follows:

(1) scope certification;
(2) kick-off meeting;
(3) system installation;
(4) LIMS workbook;
(5) utilization workshop;
(6) LIMS specification;
(7) activation tasks;
(8) training;
(9) go live;
(10) LVS alliance services.

Each step is accompanied with deliverables, which will determine progress to the next stage. Therefore, the Project Manager and the customer are synchronized throughout the installation.

### Automation of laboratory weighing processes

*Suzanne Blohm and Michael L. Ross[1], Bohdan Automation, 562 Bunker Court, Vernon Hills, IL 60061, USA; [1]Mettler-Toledo, 1900 Polaris Parkway, Columbus, OH 43240, USA*

Weighing is one the most common tasks in any laboratory. Over 100 000 analytical balances are sold annually, worldwide, and there is a balance in just about every laboratory in every type of industry. Bohdan Automation, a Mettler Toledo company, has developed a simple, cost-effective alternative to manual weighing, the balance automator. The automator has been used to automate the weighing processes being performed in the food industry, pharmaceutical industry and water industry to name a few.

Automating a weighing process improves laboratory efficiency by freeing up scientist time spent performing simple repetitive tasks. Additional advantages, e.g. error-free documentation and increased number of samples processed have been recognized.

The balance automator utilizes interchangeable parts to allow one unit to accommodate multiple container sizes, so virtually any weighing process can be automated. It is compatible with a number of balances.

The controller can be networked with a laboratory information management system (LIMS) for seamless data transfer.

Examples of how the automator has been used in the food industry, pharmaceutical industry and water industry will be presented in this paper.

### Automating synthesis support activities in combinatorial chemistry

*Ben Moshiri and Suzanne Blohm, Bohdan Automation, 562 Bunker Court, Vernon Hills, IL 60061, USA*

Typically, drug discovery includes a sequence of steps, e.g. reagent preparation, compound synthesis, compound dissolution, product distribution, and structural characterization and activity analysis. This paper describes a new workstation, developed to address time-consuming steps before and after synthesis.

Reagent preparation: using reagent data files as inputs, the operator is prompted with compound weight ranges for quick reagent weighing, followed by automatic dispensing of solvents and vortex mixing of solutions.

Compound dissolution: newly synthesized, dried compounds in collection tubes are volumetrically or gravimetrically prepared to a fixed concentration or volume and mixed by vortexing.

Product distribution: dissolved synthesis products are transferred into appropriate containers (e.g. deep-well plates, GC/LC vials) for chemical or biological screening.

### Optimization of automated solid phase extraction methods

*Michael Urban-Piette and Paul Bouis*[1]*, Gilson, Middleton, WI 53562, USA;* [1]*Mallinckrodt Baker International, Phillipsburg, NJ 08865, USA*

The accelerating trend to develop more sensitive and robust analytical methods that allow for high-throughput screening (HTS) of smaller and smaller samples is pointing many laboratories toward automation of their sample preparation steps both in method development and final product testing. The clear advantages of automation, e.g. precise and repetitive delivery of reagents, unattended operation and increased quality assurance testing become apparent when speed, sensitivity and precision are critical parameters.

Solid phase extraction (SPE) is one of the fastest growing segments of sample preparation in biotechnology and pharmaceutical analysis. Sample preparation is the rate-limiting step in most analytical methods used in HTS. Automation of SPE must be a high priority if the benefits of new drug development techniques, e.g. combinatorial chemistry are to be realized. HTS depends in part on the development of automated SPE technology that can handle the large number of small-volume samples generated on a continuous basis.

The efficiency of the sorbent used in the selective purification of analytes from complex mixtures is a critical component of any SPE method. Today's routine use of 30–60 μm particles in most SPE devices limits its usefulness. Even with the perfect sorbent, automating a bad method can quickly kill even the best project. The combined performance of an automation system and SPE using a novel extraction device which allows for the use of more efficient 10 μm particles was examined. The impact of various automation parameters on recovery, precision, linearity and throughput was included in the study. This paper will demonstrate that dividing all extraction parameters into two categories can simplify automated SPE method development: first, those that pertain solely to automation; second, those that pertain to the underlying SPE method.

### Miniature infrared spectrometer for process applications

*Suneet Chadha, William Kyle, Larry Curtiss, Chuck Stevenson and Mark Druy*[1]*, Foster-Miller, 195 Bear Hill Road, Waltham, MA 02154, USA;* [1]*Sensiv, 195 Bear Hill Road, Waltham, MA 02154, USA*

Infrared (IR) spectroscopy is routinely used in analytical laboratories and process applications, and is the basis for analysis and identification of organic compounds. Traditional FTIR instruments have been the basis of such measurements.

Foster-Miller has developed a unique small footprint spectrometer (3–12 μm) for process applications. This miniature spectrometer, which, for the intended use, has dimensions of the order of 6″ × 4″ × 2″, can equal the measurement accuracy and throughput of an FTIR. Bulk IR transmitting optics or chalcogenide fibres are used to couple a pulsed source to the spectrometer. Sampling may be performed in either transmission or reflectance modes as well as be coupled to fibre optic ATR probes. A monolithic wedge-grating optic provides the spectral dispersion with low-cost thermopile point or array detectors picking off the diffracted wavelengths from the optic. The integrated optic provides spectral discrimination with resolution at 8 $cm^{-1}$ or better, and overall optical throughput approaching 45%.

The approach is based on our proprietary miniature IR spectrometer, currently under development as an on-line/on-board lubricant condition monitoring for aircraft turbine engines. The device has a fixed cylindrical grating uniquely bonded to the edge of a ZnSe conditioning 'wedge'. The conditioning optic overcomes the limitations of concave gratings as it accepts high-angle (large FOV) light at the narrow end of the wedge and progressively conditions it to be near normal to the grating. On return, the diffracted wavelengths are concentrated on the discrete detector (pixel) elements by the wedge, providing throughput comparable to that of an FTIR.

The device will eliminate the cost, complexity, reliability and bandwidth/resolution problems associated with either Fabry Perot or Michelson interferometer-based approaches for low-cost process applications. We will present the performance and sensitivity expectations of this device and sampling methodologies that may be coupled to this spectrometer in this paper.

### Portable scanning infrared instrumentation for the analysis of airborne chemical composition rates

*Gaston Marzoratti and Andrew Szabo, Foxboro Company, Analytical Division, E. Bridgewater, MA 02333, USA*

Multiple infrared scans obtained from a portable, battery-operated instrument have a number of uses for the identification of airborne chemicals as well as for the determination of the changes in chemical species and concentrations that occur over time. Coupled with a spectral identification package that has the ability to measure qualitative and quantitative mixtures of airborne contaminants, an operator will have a powerful combination of tools to work with.

We have investigated two primary areas for the potential use of this technique.

(1) Indoor air quality (IAQ) assessment of various locations with regard to worker complaints and/or regulation compliance.
(2) The determination and measurement of the stability and/or reactivity of one or more chemicals present simultaneously in air.

To demonstrate the technique we have created three-dimensional wavelength, absorbance, time plots that will be used to demonstrate and discuss advantages and potential uses.

The potential uses discussed include the following.

(1) IAQ studies performed as a result of workers complaint may miss the actual cause of the complaint if its source is of a non-constant origin. Being able to bring instrumentation to the site of the complaint, and to monitor and collect infrared scans for an extended period of time would have significant value. The spectral identification program would offer three possible results.
    (i) Chemical(s) found. Identified with time and concentration data included.
    (ii) Chemical(s) found. Not identified with time data included.
    (iii) No chemical(s) found.
(2) Infrared instrumentation that has been configured to work in an environment with a known set of chemicals present may give inaccurate results if an additional chemical appears. Using spectral scans with user-definable chemical libraries compensates for the presence of unexpected chemicals.
(3) A variety of single and multiple chemical species are not stable in ambient air. To configure a system for the measurement of these chemicals, it is advantageous to be able to predict rates of conversion at a variety of sampling conditions. Using multiple scans allows us to 'see' the conversion from one species to another, and therefore allows us a better possibility to configure an accurate measurement matrix.

### Outlier detection and computation of quantitation limits in an analytical method validation study

*Kenneth L. Brubaker, Hugh J. O'Neill[1] and John F. Schneider[1], KLB Associates, 1440 Fawn Ct., Bolingbrook, IL 60490, USA; [1]Energy Systems Division, Argonne National Laboratory, 9700 S. Cass Ave., Chicago, IL 60439, USA*

The US Army is currently developing chronic exposure criteria to govern the clean up of chemical agent-contaminated waste streams. Typical examples of waste streams are used decontamination fluids, metal substrates and soil. The final criteria will probably lie in the low part-per-billion (ppb) range. Although the Army has existing analytical methods capable of detecting most agents at the ppb level, they may not be suitable for detecting agents at the criteria levels being established, or for the desired matrices. Therefore, alternative analytical techniques were developed to reach the anticipated more stringent requirements.

The method was validated in accordance with the USATHAMA quality assurance program (USATHAMA PAM 11-41, 1990). This program requires pre-certification of a method by using an eight-point calibration curve and specifies a six-level series of spiking experiments, on the basis of the TRL, to be carried out over four successive days.

Checking for outliers is required, and Dixon's test (the 'Q' test) is specified as the method of choice. This test is used almost universally in analytical chemistry to detect outliers. Because of the nature of the validation data, however, Stefansky's test was found to be more useful and reliable. Examples of its application are presented and discussed in this paper.

The associated Army software computes a 'certified reporting level' (CRL), which is the Army's designated quantitation limit. The method has been evaluated successfully when the CRL is less than the TRL. The calculation of the CRL involves a regression analysis of found versus target levels and a subsequent computation that assumes that the method standard deviation is constant over the range of interest. A relatively simple procedure for estimating the CRL that does not involve the assumption of constant standard deviation was derived and tested. The CRL values obtained with this procedure are approximately one-third of the magnitude of those computed by using the standard formulae. Computational procedures that do not take the variation of the standard deviation into account produce quantitation limits that misrepresent the capabilities of the analytical method at low concentrations.

### Single-button operation for your chromatography data system

*Maureen Stone and Tim Hawkins, Scientific Software, 6612 Owens Drive, Pleasanton, CA 94588, USA*

Computerized data systems have become an indispensable part of most chromatography laboratories, yet some operations retain the need for the simplicity of an integrator. Through the use of a user-customizable graphical user interface (GUI), it is possible to have the best of both worlds. A powerful chromatography data system design will be discussed where a full-featured client/server product can be easily modified by the laboratory manager to simplify system access. Once designed, a user logs onto the system with his/her password, and is presented with a dialogue where he or she simply presses the 'start' button once the sample tray is loaded. The potential for increased throughput, decreased operator error, minimized training time and increased data integrity will be discussed in detail in this paper.

The design can be simply modified without affecting the integrity of the chromatographic method, the integration of the sample or data security. Moreover, a system administrator can very easily modify it to meet the particular needs of the user.

Finally, for client/server-based chromatography data systems, a custom interface can be created for each individual client, thus allowing error-free sample analysis by non-laboratory personnel, as well as full access to all the features enjoyed by an advanced chromatographer, all in one system.

### Total chromatography data management from within a LIMS

*Ian Jennings, LabSystems, 1 St George's Court, Hanover Business Park, Altrincham, Cheshire WA14 5TP, UK*

One of the key justifications in purchasing a LIMS is usually the integration of laboratory instrumentation. Yet in practice this rarely happens, in a recent survey (SDI, LIMS in North America, June 1998), over 50% of respondents reported that none of their instruments was connected to their LIMS. This paper will examine the merits of integration, and using chromatography data systems as an example, discuss how emerging standards in technology can ease systems integration.

Over the last couple of years, there has been an overwhelming acceptance of Microsoft's operating systems and office applications the world over, and laboratories are no different. Here the de facto standards are Intel, Windows 95, Windows NT 4.0 and Office 95 & 97.

The emergence of standards, e.g. COM and OLE, together with Microsoft's hegemony of the desktop, have created an environment whereby laboratory software can work together. Linking and integrating LIMS and chromatography systems together, e.g. alongside connecting instruments to LIMS, probably ranks as the largest single area of potential productivity enhancement in most laboratories today. Chromatography is still the most commonly used analytical technique and in terms of results, generates the bulk of most laboratories output.

However, problems remain to be addressed in this area. These include dealing with missing or unknown components, unregistered samples, terminology and field mapping, and archiving chromatograms in LIMS. But with integration providing both a key productivity benefit, and a key differentiator between system suppliers, these issues are currently being considered and will be continue to be addressed in the future.

### Implementing secure electronic records and electronic signatures in a chromatography data system

*Peter W. Yendle, LabSystems R&D, 1 St George's Court, Hanover Business Park, Altrincham, Cheshire WA14 5TP, UK*

To increase productivity in the laboratory, it is highly desirable to be able to perform on-line review and authorization of chromatographic data. In an FDA-regulated environment, achieving this productivity increase requires compliance with the electronic records and electronic signatures requirements of Chapter 21 of the Code of Federal Regulations (21 CFR Part 11).

This paper presents a novel approach to meeting these requirements in a chromatography data system. It discusses the following key issues.

(1) Implications of the FDA regulations for the system developer, system administrator and chromatographer.
(2) The need for flexible configuration of the system to accommodate the varying workflow in different laboratory environments.
(3) The need for close integration with the operating system when designing a secure data system.
(4) The use of Windows NT™ as a secure operating system in the laboratory.
(5) Configuring and operating a secure chromatography data system.

### Using ASTM D6122 as a guideline for validating process NIR spectrophotometers and setting practical action limits

*Michael A. Broussard and Terry Todd, UOP Guided Wave, 5190 Golden Foothill Parkway, El Dorado Hills, CA 95762, USA*

As a result of the approval of ASTM D6122, Standard Practice for Validation of Multivariate Process Infrared Spectrophotometers, supporters of process NIR technology have been supplied with a means to routinely verify hardware performance—in easy to understand terms. Until now, the scarcity of universally accepted performance criteria has often left the spectrophotometer as an easy target when predicted values conflict with either lab results or familiar processing patterns. Troubleshooting activities to resolve these conflicts often lead investigators to the laboratory, the modeller, and ultimately to the spectrometer. Because the inanimate instrument is incapable of debating its position, it is frequently deemed the culprit. In the worst of cases, the instrument is taken off line permanently. This action is very costly. Losses include the substantial initial investment of purchase and also the forgone opportunity to realize any speculated benefits.

The ASTM standard proposes three levels of testing for NIR process spectrometers that provides a 'voice' for the instrument. One particular D6122 testing level called Level 0 describes five specific parameters that may be used to effectively assess spectrophotometer performance. Other guidelines and recommendations are also present for setting up an analyser validation program that is both adequate and easy to attain.

In concept, a stable, translucent sample is used as a standard and is scanned by the instrument beam. The Level 0 parameters are calculated. Current data are then referenced against the historical performance data. The analyser is assigned to 'valid' or 'invalid' status based on true site performance requirements instead of arbitrary factory settings. Therefore, choosing action limits for control becomes a function of experience and model sensitivities, and thus may be considered more practical.

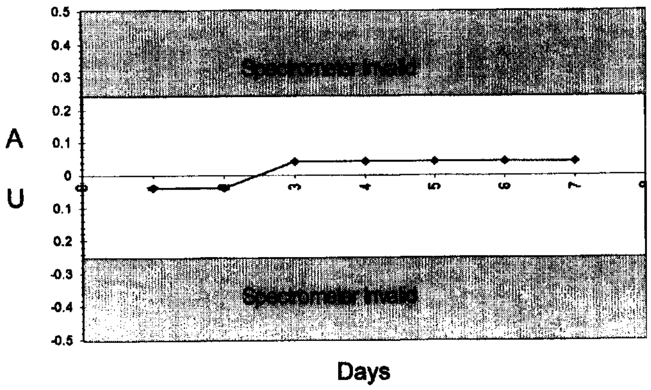

*Level 0 control chart for spectral baseline stability.*

### Total software solution for remote monitoring of process NIR analysers

*Alagappan Annamalai, Howard D. Alpert and Richard E. Mansker, UOP LLC, Guided Wave, 5190 Golden Foothill Parkway, El Dorado Hills, CA 95762, USA*

Every year, as more NIR analysers are incorporated in real-time monitoring of chemical processes, chemometricians and plant engineers become responsible for more analysers. As a result, human–analyser interaction from remote locations is becoming a necessity rather than convenience to manage these multiple systems properly. We have developed a software product with a client-server architecture that provides a client human–analyzer interface from virtually anywhere via local or wide area networks. This multi-tasking software was designed for widely used Windows NT and Windows 95 operating systems.

Features included in the product provide a total software solution for typical process NIR analyser problems. For example, the software provides programmable interfaces to MODBUS® protocol and OPTOMUX® device. Through these devices and protocols, the analytical results and their validation statistics can be transmitted to a distributed control system (DCS). This information is necessary for DCS in making proper process control decisions. The software has the capabilities to periodically collect analyser diagnostic data and download spectral files to a remote location without human intervention. In process NIR analysis, managing calibration files, prediction algorithms, primary data values, etc. for multiple parameters and multiple spectra is often a cumbersome error-prone task. In this software, we propose new concepts to manage these data easily. For example, we pack model information for multiple parameters together in one file, and also keep the primary data with the corresponding sample spectrum together in a file. We will discuss the concepts, architecture and advantages of this software in this paper.

### Pulse introduction membrane extraction (PIME) for fast on-line extraction of volatile organics from an aqueous matrix

*X. Guo, A. Sanjuan and S. Mitra, Department of Chemical Engineering, Chemistry New Jersey Institute of Technology, Newark, NJ 07102, USA*

Most analytical applications of membrane extraction have involved continuous introduction of the sample into the membrane. In these studies, the measurements were made after membrane permeation reached a steady state. Because the diffusion of analytes in the aqueous matrix and through the membrane is a slow process, it takes a certain amount of time to reach steady state. Any measurement made during the unsteady state period does not represent the true concentration of the sample stream. Furthermore, a relatively large sample volume is needed for the analysis because the sample has to be introduced continuously. Recently we have developed an alternative approach to membrane extraction referred to as pulse introduction membrane extraction (PIME). Here, a pulse of sample is injected into the membrane, and steady state is not reached. This approach results in an analytical system that has a faster response and the capability to analyse individual samples. This concept can be used in conjunction with gas chromatography, mass spectrometry, as well as other analytical techniques.

In this paper, the application of PIME for analysis of individual samples and continuous monitoring of trace level organics in water is presented. The system demonstrated sub ppb level detection limits, high precision and linear calibration curves. In this study, conditions for shorter analysis time and high sensitivity were studied. A comparison of PIME with continuous sample introduction is also presented.

